# Supplementary material for: Asymmetric gradient orbital interaction of hetero-diatomic active sites for promoting C − C coupling
Source: Nat Commun. 2023 Jun 27;14:3808. doi: 10.1038/s41467-023-39580-5 (PMC10300110; doi:10.1038/s41467-023-39580-5)
Supplement: Supplementary file 1 — Supplementary Information [file 41467_2023_39580_MOESM1_ESM.pdf]

Asymmetric gradient orbital interaction of hetero-diatomic active sites for promoting C−C coupling

Jin Ming Wang[1], Qin Yao Zhu[1], Jeong Heon Lee[1], Tae Gyun Woo[1], Yue Xing Zhang[2], Woo-Dong Jang[1], and Tae Kyu Kim*[1]

[1]Department of Chemistry, Yonsei University, Seoul 03722, Republic of Korea

[2]College of Chemistry and Chemical Engineering, Dezhou University, Dezhou 253023, China

Correspondence to: tkkim@yonsei.ac.kr (T. K. Kim)

Supplementary Methods

General characterizations

Powder X-ray diffraction (PXRD) patterns were obtained on MiniFlex 600 diffractometer with Cu K_α (λ = 0.154 nm) focused radiation at 40 kV and 40 mA. High-angle annular dark-field or bright-field scanning transmission electron microscopy (HAADF or BF-STEM) characterization and corresponding energy-dispersive spectroscopy (EDS) were conducted on holy Au grids at a JEOL JEM-ARF200F TEM/STEM system with a spherical aberration corrector under 200 kV Schottky cold-field emission gun. UV-Vis diffuse reflectance absorption spectra (DRS) were recorded at ambient temperature on UV1800 SHIMADZU. X-ray photoelectron spectra (XPS) were conducted on an ESCALAB 250Xi X-ray photoelectron spectroscope (Thermo Fisher, Al K_α, 1486.6 eV), and all samples were followed an Ar etching process before XPS analysis. Picosecond time-resolved photoluminescence (TRPL) spectra were measured on a femtosecond fluorescence spectrophotometer (Edinburgh FES 920). The femtosecond transient absorption setup was based on a regenerative amplified Ti:sapphire laser system from Coherent (800 nm, 35 fs, 6 μJ pulse^{-1}, and 1 kHz repetition rate), nonlinear frequency mixing techniques and the Femto-TA100 spectrometer (Time-Tech Spectra). The Zn, Ru, and Cu contents were determined by inductively coupled plasma atomic emission spectroscopy (ICP-AES) analysis with an IRIS Intrepid II XRP instrument. Under an ultrahigh vacuum (UHV) chamber, ultraviolet photoelectron spectroscopy (UPS) was obtained by synchrotron radiation photoemission spectroscopy (SRPES) measurements. The valence-band spectra were measured using synchrotron-radiation light as the excitation source with photon energy of 40.00 eV and referenced to the Fermi level (E_F = 0) determined from Au. A sample bias of –5 V was applied in order to observe the secondary electron cutoff. The work function (WF) is determined by the difference between the photon energy and the binding energy of secondary cutoff edge (WF = hv – (E_{cutoff} – E_F), hv: photo energy,

E_{cutoff}: secondary cutoff edge, and E_F: Fermi level). X-ray absorption spectroscopy (XAS), including both X-ray absorption near-edge structure (XANES) and extended X-ray absorption fine structure (EXAFS) at Zn, Ru and Cu K-edge (or L_3-edge) were collected in total-fluorescence-yield mode at ambient air at the Nano-crystallography and Coherence Imaging Experimental Station with the BL01C1 beamline facility. EXAFS analysis was conducted using Fourier transform on $\mathbf{k}^3$-weighted EXAFS oscillations to evaluate the contribution of each bond to the Fourier transform peak.

The quasi in-situ Raman spectroscopy

The quasi in-situ Raman measurements were carried out on a confocal microscope Raman system (XploRA, HORIBA). A He-Ne laser with 630 nm excitation wavelength and a 50× microscope objective with a numerical aperture of 0.55 was used in all measurements. Raman frequency was calibrated by a Si wafer during each experiment. In situ chemical Raman experiments were employed in a homemade Raman cell, where the photocatalyst was dispersed in the same ingredient and concentration as CO_2 photoreduction experiment. After the quasi in-situ Raman system of ZnPor-RuCuDAC was thoroughly cleansed by Ar gas in 30 min, pure CO_2 was purged into the chamber, intermittently collecting the quasi in-situ Raman signal from 0 to 60 min until CO_2 adsorption saturation. For CO_2 photoreduction in-situ Raman measurements, the sample was illuminated by a 150W Xe-lamp (Abet Technologies). In order to evade the interference of Xe lamp light on the detection laser, it should be mentioned that the Xe lamp is turned off for a short time when collecting the spectrum.

The in-situ diffuse reflectance infrared Fourier transform spectra

With different irradiation time, in-situ diffuse reflectance infrared Fourier transform spectra (DRIFTS) of these COFs sample was obtained on Thermo Electron Nicolet iS50 spectrometer, equipped with liquid N_2 cooled HgCdTe (MCT/A) detector. In a typical process, 2 mL prepared COF samples, containing the same ingredient and

concentration as CO_2 photoreduction experiment, were firstly dispersed on the DRIFTS accessory (two ZnSe windows and one SiO_2 window), and then degassed at 120 °C for 6 h. Typical signals of various intermediates were captured subsequently after the introduction of the flowed CO_2 and H_2O vapors under dark and light irradiation (0 – 80 min). To explore the influence of diatomic COF catalysts on the chemisorbed *CO, in-situ DRIFTS of CO gas adsorption were measured. In a typical process, the catalyst film was put in the accessory, which was sealed and purged with N_2 for 20 min. Then CO gas was switched into the system to collect the CO adsorption signal until equilibrium. After purging N_2 again, the CO desorption signals were captured from 0 to 90 min. Therefore, the strongest peak is assigned to CO adsorption and desorption, and the peak intensity gradually decreased during N_2 purging to remove the adsorbed CO as time extension.

Isotope labeling measurement

The isotope labeling measurement was carried out by using $^{13}CO_2$ gas (isotope purity, 99%) instead of pure $^{12}CO_2$ gas (Chemical purity, 99.999%) as the carbon source with the same photocatalytic process, and the reaction was conducted for 6 h. The photocatalytic species were separated by gas spectrometry columns into individual substances for detecting the products of ^{13}CO (HP-MOLESIEVE column, USA) and formate/acetate acids (HP-FFAP column, USA). As the separated substances emerge from the column, they flow into electron ionization equipment to ionize and fragment analyte molecules. The separated gas products were analyzed by mass spectrometry (JMS-K9, JEOL-GCQMS, USA). The helium was used as carrier gas with a flow rate of 0.8 ml L^{-1}. The mass-to-charge (m/z) ratio of mass scanning mode were set from 2 to 70.

Computational details

The first-principle calculation was performed using the Vienna Ab-Initio Simulation Package based on spin-polarized Perdew–Burke–Ernzerhof functional. Van der Waals correction of Grimme scheme (D2) was used to improve the description of the dispersion interaction between adsorbates and substrates. A vacuum thickness of over 15 Å was added in the z direction to avoid unphysical interactions between periodic images. In order to ensure accurate results, all calculations were conducted with a plane wave cutoff of 500 eV and a 2 × 2 × 1 Monkhorst-Pack *k*-point. In order to elucidate the structures of these COFs and calculate unit cell parameters, three types of possible 2D structure were generated for ZnPor-RuCuDAC (AA-eclipsed, AB-staggered, and slipped ABC-staggered stacking models) by using the density-functional tight-binding (DFTB) method. Pawley refinement was carried out by using Reflex package, which was a commercial-free software package for crystallographic structural analysis from PXRD pattern, implemented in Material Studio 2019 modeling version. Unit cell dimensions were manually resolved from the obtained PXRD pattern positions using the coordinates. Then Pawley refinement was carried out to optimize the lattice parameters iteratively until the R_{WP} values converged and the observed overlay with refined profiles showed good agreements.

Moreover, the possible reduction path for these COFs catalytic systems could be as follows, where the asterisks denote active sites and the vertical arrows represent the produced gas in the intermediate reaction.

First possible reduction pathways:

$^{*} + CO_2 + e^- + H^+ \rightarrow {}^{*}COOH$ (1)

Or $^{*}COOH + e^- + H^+ \rightarrow {}^{*}HCOOH$ $\quad {}^{*}HCOOH \rightarrow {}^{*} + HCOOH$ (2-1)

Or $^{*}COOH + e^- + H^+ \rightarrow {}^{*}CO + H_2O$ $\quad {}^{*}CO \rightarrow {}^{*} + CO\uparrow$ (2-2)

$^{*}CO + {}^{*} + CO_2 + e^- + H^+ \rightarrow {}^{*}CO + {}^{*}COOH$ (3)

$^{*}CO + {}^{*}COOH + e^- + H^+ \rightarrow {}^{*}CO + {}^{*}CO + H_2O$ (4)

$^{*}CO + {}^{*}CO \rightarrow {}^{*}OC–CO$ (5)

$^{*}OC–CO + e^- + H^+ \rightarrow {}^{*}OC–COH$ (6)

$^*OC–COH + e^– + H^+ \rightarrow {}^*OC–CHOH$ (7)

$^*OC–CHOH \rightarrow {}^*OC–O–CH_2$ (8)

$^*OC–O–CH_2 + e^– + H^+ \rightarrow {}^*HOC–O–CH_2$ (9)

$^*HOC–O–CH_2 \rightarrow {}^*HO(COC)H_2$ (10)

$^*HO(COC)H_2 + e^– + H^+ \rightarrow {}^*HOOC–CH_3$ (11)

$^*HOOC–CH_3 \rightarrow {}^* + HOOC–CH_3$ (12)

Second possible reduction pathways:

$^* + CO_2 + e^– + H^+ \rightarrow {}^*COOH$ (13)

or $^*COOH + e^– + H^+ \rightarrow {}^*HCOOH$ $^*HCOOH \rightarrow {}^* + HCOOH$ (14-1)

or $^*COOH + e^– + H^+ \rightarrow {}^*CO + H_2O$ $^*CO \rightarrow {}^* + CO\uparrow$ (14-2)

$^*CO + {}^* + CO_2 + e^– + H^+ \rightarrow {}^*CO + {}^*COOH$ (15)

$^*CO + {}^*COOH + e^– + H^+ \rightarrow {}^*CO + {}^*CO + H_2O$ (16)

$^*CO + {}^*CO \rightarrow {}^*OC–CO$ (17)

$^*OC–CO + e^– + H^+ \rightarrow {}^*OC–COH$ (18)

$^*OC–COH + e^– + H^+ \rightarrow {}^*OC–CHOH$ (19)

$^*OC–CHOH + e^– + H^+ \rightarrow {}^*OC–CH + H_2O$ (20)

$^*OC–CH + e^– + H^+ \rightarrow {}^*OC–CH_2$ (21)

$^*OC–CH_2 + H_2O \rightarrow {}^*(HO)_2C–CH_2$ (22)

$^*(HO)_2C–CH_2 \rightarrow {}^*HOOC–CH_3$ (23)

$^*HOOC–CH_3 \rightarrow {}^* + HOOC–CH_3$ (24)

Gibbs free energies of each intermediate were calculated at 298.15 K from $G = E_{DFT} + E_{ZPE} – TS$, where E_{DFT} is the electronic energy, E_{ZPE} is the zero-point energy, and TS is the entropy contribution. Standard ideal gas methods were employed to compute E_{ZPE} and TS from temperature, pressure, and the calculated vibrational energies. In particular, $G[e^- + H^+] = 1/2G[H_2] – eU$, where U is the applied overpotential and e is the elementary charge. We set U = 0 V vs. reversible hydrogen electrode. The free energy changes (ΔG) relative to an initial state can be obtained by the difference between products and reactants at every step.

Supplementary Figures and Tables

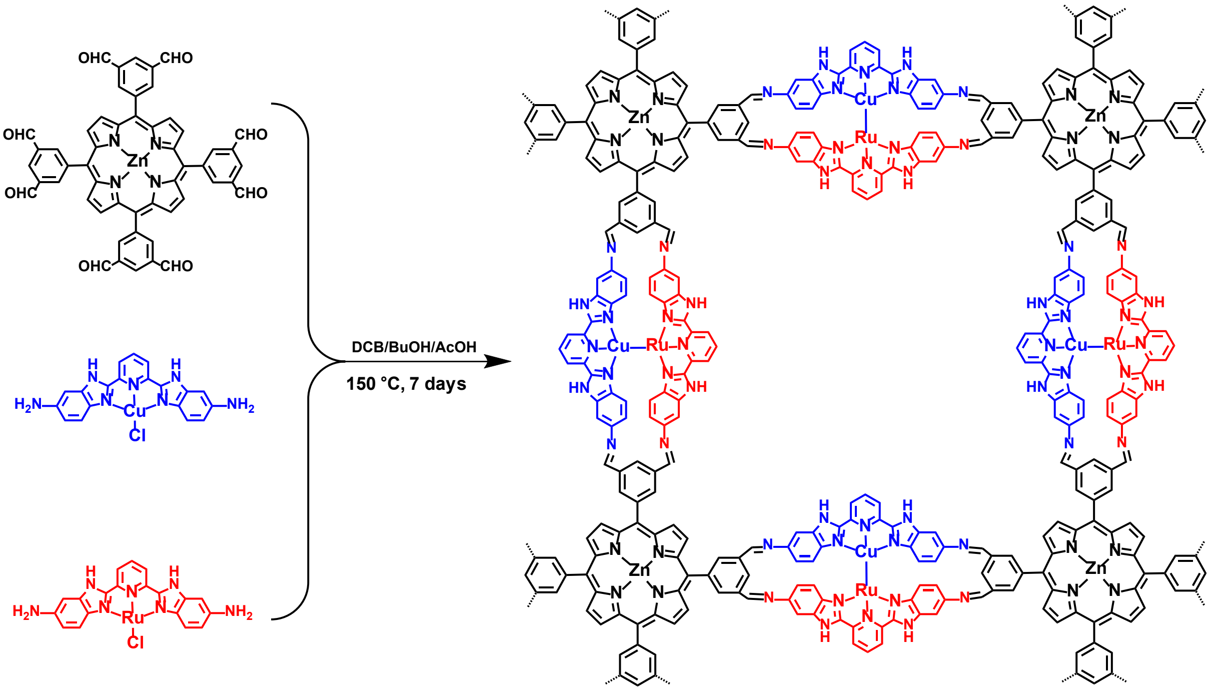

Supplementary Figure 1. Reaction routes for the synthesis of ZnPor-RuCuDAC COF.

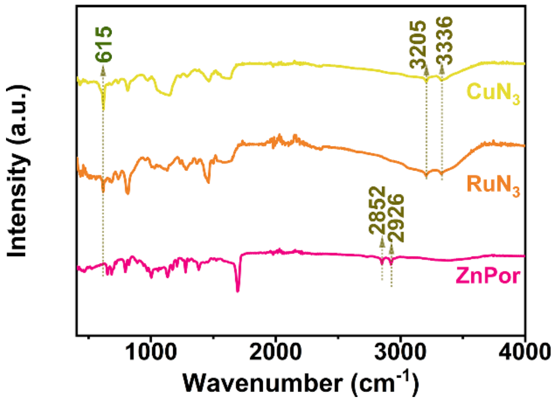

Supplementary Figure 2. FTIR spectra of ZnPor, RuN_3, and CuN_3 monomers.

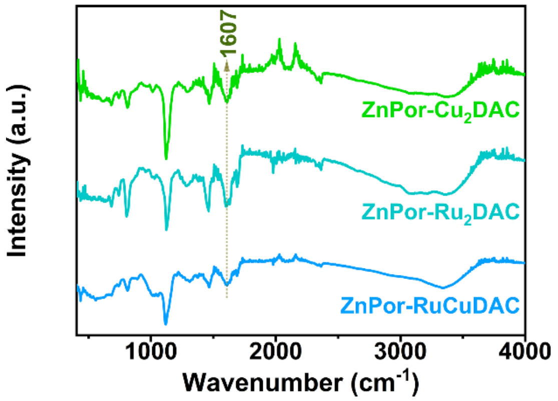

Supplementary Figure 3. FTIR spectra of ZnPor-RuCuDAC, ZnPor-Ru_2DAC, and ZnPor-Cu_2DAC COF.

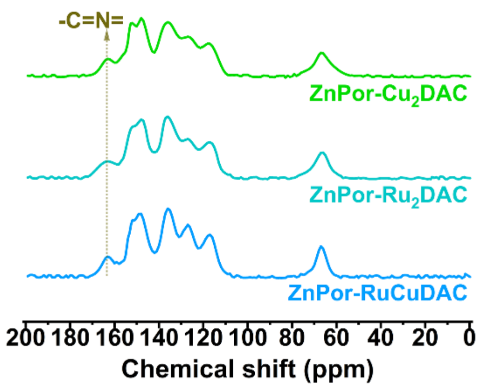

Supplementary Figure 4. Solid-state ^{13}C NMR spectra of ZnPor-RuCuDAC, ZnPor-Ru_2DAC, and ZnPor-Cu_2DAC COF.

Supplementary Table 1. The contents of Zn, Ru, and Cu elements presented in Zn-COF, Cu-COF, and ZnCu-COF determined by ICP-OES.

COF	C(%)[a)]	H(%)[a)]	N(%)[a)]	Zn(%)[b)]	Ru(%)[b)]	Cu(%)[b)]
ZnPor-RuCuDAC[c)]	62.72	3.02	18.16	2.62	8.29	5.20
	(62.67)	(2.96)	(18.28)	(2.67)	(8.24)	(5.18)
$ZnPor-Ru_2DAC$[c)]	60.76	2.83	17.60	2.63	16.18	0
	(60.81)	(2.87)	(17.73)	(2.59)	(16.00)	(0)
$ZnPor-Cu_2DAC$[c)]	64.70	3.13	18.73	2.71	0	10.73
	(64.65)	(3.05)	(18.85)	(2.76)	(0)	(10.69)

[a)] Data calculated from element analysis results.

[b)] Data determined with ICP-OES.

[c)] Data in parentheses were theoretically values calculated according to the following formula.

Note: The stoichiometric ratio of precursor monomers (ZnPor, RuN_3, and CuN_3) will be tightly controlled when the COF coupling reaction terminates. Because it will take off 8eq of H_2O and 4eq of Cl if ZnPor is 1eq in this process, the theoretical elemental values were calculated by using the following formula: 1) ZnPor-RuCuDAC [1×(ZnPor) + 2×(RuN_3) + 2×(CuN_3) – 8×(H_2O) – 4×Cl)]/5 = $C_{25.6}H_{14.4}N_{6.4}Zn_{0.2}Ru_{0.4}Cu_{0.4}$; 2) ZnPor-$Ru_2$DAC [1×(ZnPor) + 4×($RuN_3$) – 8×($H_2O$) – 4×Cl)]/5 = $C_{25.6}H_{14.4}N_{6.4}Zn_{0.2}Ru_{0.8}$; 3) ZnPor-$Cu_2$DAC [1×(ZnPor) + 4×($RuN_3$) – 8×($H_2O$) – 4×Cl)]/5 = $C_{25.6}H_{14.4}N_{6.4}Zn_{0.2}Cu_{0.8}$.

Supplementary Table 2. Fractional atomic coordinates for AA-eclipsed ZnPor-RuCuDAC unit cells.

Type	Fractional	coordinate	
C1	0.3251	0.49999	0.07631
C2	0.31015	0.49999	0.09154
C3	0.32139	0.49999	0.11198
N4	0.34197	0.49999	0.10869
C5	0.34568	0.49999	0.08647
C6	0.4203	0.49999	0.09209
C7	0.40425	0.49999	0.07645
C8	0.38532	0.49999	0.08605
N9	0.38943	0.49999	0.10745
C10	0.41097	0.49999	0.11198
C11	0.36461	0.49999	0.07604
C12	0.40397	0.49999	0.1866
C13	0.41893	0.49999	0.17138
C14	0.40836	0.49999	0.1508
N15	0.38628	0.49999	0.15505
C16	0.38326	0.49999	0.17563
C17	0.4214	0.49999	0.13173
C18	0.31084	0.49999	0.17234
C19	0.32606	0.49999	0.18729
C20	0.34732	0.49999	0.17673
N21	0.34238	0.49999	0.15464
C22	0.32099	0.49999	0.1508
C23	0.31042	0.49999	0.13187
C24	0.36351	0.49999	0.18688
C25	0.44376	0.49999	0.13173
C26	0.2882	0.49999	0.13201

C27	0.36392	0.49999	0.20924
C28	0.36406	0.49999	0.05368
C29	0.45487	0.49999	0.15107
C30	0.47723	0.49999	0.15107
C31	0.48834	0.49999	0.13173
C32	0.47723	0.49999	0.11239
C33	0.45487	0.49999	0.11239
C34	0.27695	0.49999	0.11267
C35	0.25473	0.49999	0.11267
C36	0.24348	0.49999	0.13201
C37	0.25473	0.49999	0.15135
C38	0.27695	0.49999	0.15135
C39	0.34485	0.49999	0.2209
C40	0.34485	0.49999	0.24312
C41	0.36392	0.49999	0.25382
C42	0.38299	0.49999	0.24312
C43	0.38299	0.49999	0.2209
C44	0.38299	0.49999	0.04175
C45	0.38299	0.49999	0.01952
C46	0.36406	0.49999	0.0091
C47	0.34526	0.49999	0.0209
C48	0.34526	0.49999	0.04326
C49	0.33415	0.49999	0.25807
C50	0.24678	0.49999	0.16739
C51	0.24472	0.49999	0.09771
C52	0.39848	0.49999	0.25725
Zn53	0.36667	0.49999	0.13434
C54	0.42237	0.60997	0.36438

C55	0.42296	0.6106	0.39592
C56	0.44059	0.64493	0.39558
C57	0.44917	0.66191	0.37964
C58	0.44	0.6443	0.36404
N59	0.41392	0.59325	0.38032
C60	0.39233	0.55258	0.29623
C61	0.37727	0.52307	0.30557
C62	0.37761	0.52344	0.32382
C63	0.39302	0.55331	0.3327
C64	0.40808	0.58281	0.32322
C65	0.40774	0.58244	0.30498
N66	0.39706	0.5609	0.35047
C67	0.41458	0.595	0.35191
N68	0.42143	0.60861	0.33517
C69	0.38012	0.52614	0.45794
C70	0.39553	0.556	0.4667
C71	0.41059	0.5855	0.45735
C72	0.41025	0.58514	0.43911
C73	0.39484	0.55527	0.43022
C74	0.37977	0.52577	0.43971
N75	0.42314	0.61045	0.42665
C76	0.41567	0.59617	0.41006
N77	0.39821	0.56214	0.41231
N78	0.39182	0.55644	0.49001
N79	0.39055	0.55214	0.27428
Cu80	0.38134	0.52977	0.38205
C81	0.30972	0.38996	0.40073
C82	0.30913	0.38933	0.36918

C83	0.29151	0.355	0.36953
C84	0.28292	0.33802	0.38548
C85	0.2921	0.35563	0.40108
N86	0.31817	0.40668	0.38479
C87	0.33976	0.44735	0.46889
C88	0.35496	0.47711	0.45953
C89	0.35462	0.47675	0.44129
C90	0.33908	0.44662	0.4324
C91	0.32401	0.41712	0.44188
C92	0.32436	0.41749	0.46013
N93	0.33516	0.43929	0.41464
C94	0.31751	0.40493	0.41307
N95	0.31067	0.39132	0.42994
C96	0.35198	0.4738	0.30716
C97	0.33657	0.44393	0.2984
C98	0.3215	0.41443	0.30776
C99	0.32184	0.41479	0.32599
C100	0.33725	0.44466	0.33489
C101	0.35232	0.47416	0.32541
N102	0.30895	0.38949	0.33846
C103	0.31642	0.40376	0.35505
N104	0.33401	0.43805	0.35279
N105	0.3389	0.44349	0.27646
N106	0.34154	0.4478	0.49083
Ru107	0.35088	0.47042	0.38306

Supplementary Table 3. Fractional atomic coordinates for AB-staggered ZnPor-RuCuDAC unit cells.

Type	Fractional	coordinate	
C1	0.33196	0.24974	0.07635
C2	0.317	0.24974	0.09158
C3	0.32825	0.24974	0.11201
N4	0.34883	0.24974	0.10872
C5	0.35253	0.24974	0.0865
C6	0.42716	0.24974	0.09212
C7	0.41111	0.24974	0.07649
C8	0.39218	0.24974	0.08609
N9	0.39629	0.24974	0.10749
C10	0.41783	0.24974	0.11201
C11	0.37146	0.24974	0.07608
C12	0.41083	0.24974	0.18664
C13	0.42578	0.24974	0.17141
C14	0.41522	0.24974	0.15083
N15	0.39314	0.24974	0.15509
C16	0.39012	0.24974	0.17566
C17	0.42825	0.24974	0.13177
C18	0.31769	0.24974	0.17237
C19	0.33292	0.24974	0.18732
C20	0.35418	0.24974	0.17676
N21	0.34924	0.24974	0.15468
C22	0.32784	0.24974	0.15083
C23	0.31728	0.24974	0.1319
C24	0.37037	0.24974	0.18691
C25	0.45061	0.24974	0.13177

C26	0.29506	0.24974	0.13204
C27	0.37078	0.24974	0.20927
C28	0.37091	0.24974	0.05372
C29	0.46172	0.24974	0.15111
C30	0.48408	0.24974	0.15111
C31	0.49519	0.24974	0.13177
C32	0.48408	0.24974	0.11243
C33	0.46172	0.24974	0.11243
C34	0.28381	0.24974	0.1127
C35	0.26159	0.24974	0.1127
C36	0.25034	0.24974	0.13204
C37	0.26159	0.24974	0.15138
C38	0.28381	0.24974	0.15138
C39	0.35171	0.24974	0.22093
C40	0.35171	0.24974	0.24315
C41	0.37078	0.24974	0.25385
C42	0.38984	0.24974	0.24315
C43	0.38984	0.24974	0.22093
C44	0.38984	0.24974	0.04178
C45	0.38984	0.24974	0.01956
C46	0.37091	0.24974	0.00913
C47	0.35212	0.24974	0.02093
C48	0.35212	0.24974	0.04329
C49	0.34101	0.24974	0.25811
C50	0.25363	0.24974	0.16743
C51	0.25157	0.24974	0.09775
C52	0.40534	0.24974	0.25728
Zn53	0.37352	0.24974	0.13437

C54	0.42923	0.30473	0.36441
C55	0.42982	0.30505	0.39596
C56	0.44744	0.32221	0.39562
C57	0.45602	0.3307	0.37967
C58	0.44685	0.3219	0.36407
N59	0.42078	0.29637	0.38036
C60	0.39919	0.27604	0.29626
C61	0.38412	0.26128	0.30561
C62	0.38446	0.26147	0.32385
C63	0.39987	0.2764	0.33274
C64	0.41494	0.29115	0.32326
C65	0.4146	0.29097	0.30502
N66	0.40392	0.2802	0.3505
C67	0.42144	0.29725	0.35194
N68	0.42828	0.30405	0.33521
C69	0.38697	0.26282	0.45798
C70	0.40238	0.27775	0.46674
C71	0.41745	0.2925	0.45739
C72	0.41711	0.29232	0.43915
C73	0.4017	0.27738	0.43026
C74	0.38663	0.26263	0.43974
N75	0.43	0.30497	0.42669
C76	0.42253	0.29783	0.41009
N77	0.40507	0.28082	0.41235
N78	0.39868	0.27797	0.49005
N79	0.39741	0.27582	0.27432
Cu80	0.3882	0.26463	0.38209
C81	0.31658	0.19473	0.40077

C82	0.31599	0.19441	0.36922
C83	0.29837	0.17725	0.36957
C84	0.28978	0.16876	0.38551
C85	0.29896	0.17756	0.40111
N86	0.32503	0.20309	0.38483
C87	0.34662	0.22342	0.46892
C88	0.36182	0.2383	0.45957
C89	0.36148	0.23812	0.44133
C90	0.34594	0.22306	0.43244
C91	0.33087	0.20831	0.44192
C92	0.33121	0.20849	0.46017
N93	0.34202	0.21939	0.41468
C94	0.32437	0.20221	0.4131
N95	0.31752	0.19541	0.42997
C96	0.35883	0.23665	0.3072
C97	0.34343	0.22171	0.29844
C98	0.32836	0.20696	0.30779
C99	0.3287	0.20714	0.32603
C100	0.34411	0.22208	0.33493
C101	0.35918	0.23683	0.32544
N102	0.31581	0.19449	0.33849
C103	0.32328	0.20163	0.35509
N104	0.34087	0.21877	0.35282
N105	0.34576	0.22149	0.2765
N106	0.3484	0.22365	0.49086
Ru107	0.35774	0.23496	0.38309

Supplementary Table 4. Fractional atomic coordinates for slipped ABC-1 ZnPor-RuCuDAC unit cells.

Type	Fractional	coordinate	
C1	0.3251	0.16666	0.07631
C2	0.31015	0.16666	0.09154
C3	0.32139	0.16666	0.11198
N4	0.34197	0.16666	0.10869
C5	0.34568	0.16666	0.08647
C6	0.4203	0.16666	0.09209
C7	0.40425	0.16666	0.07645
C8	0.38532	0.16666	0.08605
N9	0.38943	0.16666	0.10745
C10	0.41097	0.16666	0.11198
C11	0.36461	0.16666	0.07604
C12	0.40397	0.16666	0.1866
C13	0.41893	0.16666	0.17138
C14	0.40836	0.16666	0.1508
N15	0.38628	0.16666	0.15505
C16	0.38326	0.16666	0.17563
C17	0.4214	0.16666	0.13173
C18	0.31084	0.16666	0.17234
C19	0.32606	0.16666	0.18729
C20	0.34732	0.16666	0.17673
N21	0.34238	0.16666	0.15464
C22	0.32099	0.16666	0.1508
C23	0.31042	0.16666	0.13187
C24	0.36351	0.16666	0.18688
C25	0.44376	0.16666	0.13173

C26	0.2882	0.16666	0.13201
C27	0.36392	0.16666	0.20924
C28	0.36406	0.16666	0.05368
C29	0.45487	0.16666	0.15107
C30	0.47723	0.16666	0.15107
C31	0.48834	0.16666	0.13173
C32	0.47723	0.16666	0.11239
C33	0.45487	0.16666	0.11239
C34	0.27695	0.16666	0.11267
C35	0.25473	0.16666	0.11267
C36	0.24348	0.16666	0.13201
C37	0.25473	0.16666	0.15135
C38	0.27695	0.16666	0.15135
C39	0.34485	0.16666	0.2209
C40	0.34485	0.16666	0.24312
C41	0.36392	0.16666	0.25382
C42	0.38299	0.16666	0.24312
C43	0.38299	0.16666	0.2209
C44	0.38299	0.16666	0.04175
C45	0.38299	0.16666	0.01952
C46	0.36406	0.16666	0.0091
C47	0.34526	0.16666	0.0209
C48	0.34526	0.16666	0.04326
C49	0.33415	0.16666	0.25807
C50	0.24678	0.16666	0.16739
C51	0.24472	0.16666	0.09771
C52	0.39848	0.16666	0.25725
Zn53	0.36667	0.16666	0.13434

C54	0.42237	0.20332	0.36438
C55	0.42296	0.20353	0.39592
C56	0.44059	0.21498	0.39558
C57	0.44917	0.22064	0.37964
C58	0.44	0.21477	0.36404
N59	0.41392	0.19775	0.38032
C60	0.39233	0.18419	0.29623
C61	0.37727	0.17436	0.30557
C62	0.37761	0.17448	0.32382
C63	0.39302	0.18444	0.3327
C64	0.40808	0.19427	0.32322
C65	0.40774	0.19415	0.30498
N66	0.39706	0.18697	0.35047
C67	0.41458	0.19833	0.35191
N68	0.42143	0.20287	0.33517
C69	0.38012	0.17538	0.45794
C70	0.39553	0.18533	0.4667
C71	0.41059	0.19517	0.45735
C72	0.41025	0.19505	0.43911
C73	0.39484	0.18509	0.43022
C74	0.37977	0.17526	0.43971
N75	0.42314	0.20348	0.42665
C76	0.41567	0.19872	0.41006
N77	0.39821	0.18738	0.41231
N78	0.39182	0.18548	0.49001
N79	0.39055	0.18405	0.27428
Cu80	0.38134	0.17659	0.38205
C81	0.30972	0.12999	0.40073

C82	0.30913	0.12978	0.36918
C83	0.29151	0.11833	0.36953
C84	0.28292	0.11267	0.38548
C85	0.2921	0.11854	0.40108
N86	0.31817	0.13556	0.38479
C87	0.33976	0.14912	0.46889
C88	0.35496	0.15904	0.45953
C89	0.35462	0.15892	0.44129
C90	0.33908	0.14887	0.4324
C91	0.32401	0.13904	0.44188
C92	0.32436	0.13916	0.46013
N93	0.33516	0.14643	0.41464
C94	0.31751	0.13498	0.41307
N95	0.31067	0.13044	0.42994
C96	0.35198	0.15793	0.30716
C97	0.33657	0.14798	0.2984
C98	0.3215	0.13814	0.30776
C99	0.32184	0.13826	0.32599
C100	0.33725	0.14822	0.33489
C101	0.35232	0.15805	0.32541
N102	0.30895	0.12983	0.33846
C103	0.31642	0.13459	0.35505
N104	0.33401	0.14602	0.35279
N105	0.3389	0.14783	0.27646
N106	0.34154	0.14927	0.49083
Ru107	0.35088	0.15681	0.38306
C108	0.13512	0.20354	0.18954
C109	0.10357	0.20354	0.18954

C110	0.10343	0.21501	0.20716
C111	0.11934	0.22079	0.21604
C112	0.13512	0.21501	0.20716
N113	0.11934	0.19784	0.1808
C114	0.20383	0.18481	0.16079
C115	0.19479	0.17489	0.14555
C116	0.17654	0.17489	0.14555
C117	0.16735	0.18481	0.16079
C118	0.1764	0.19474	0.17602
C119	0.19479	0.19474	0.17602
N120	0.14952	0.18723	0.1645
C121	0.1476	0.19862	0.18199
N122	0.16433	0.20328	0.18915
C123	0.04238	0.17489	0.14555
C124	0.03333	0.18481	0.16079
C125	0.04238	0.19474	0.17602
C126	0.06063	0.19474	0.17602
C127	0.06982	0.18481	0.16079
C128	0.06063	0.17489	0.14555
N129	0.07283	0.20328	0.18915
C130	0.08944	0.19862	0.18199
N131	0.08765	0.18723	0.1645
N132	0.01138	0.18481	0.15942
N133	0.22579	0.18481	0.16079
Ru134	0.11824	0.17662	0.1482
C135	0.10096	0.12977	0.07626
C136	0.13251	0.12977	0.07626
C137	0.13251	0.1183	0.05863

C138	0.11673	0.1126	0.04989
C139	0.10096	0.1183	0.05863
N140	0.11673	0.13547	0.085
C141	0.03209	0.14849	0.10501
C142	0.04129	0.1585	0.12038
C143	0.05953	0.1585	0.12038
C144	0.06872	0.14849	0.10501
C145	0.05953	0.13857	0.08977
C146	0.04129	0.13857	0.08977
N147	0.08642	0.14616	0.10143
C148	0.08834	0.13469	0.08381
N149	0.07174	0.13003	0.07665
C150	0.19369	0.15841	0.12025
C151	0.20274	0.14849	0.10501
C152	0.19369	0.13857	0.08977
C153	0.1753	0.13857	0.08977
C154	0.16625	0.14849	0.10501
C155	0.17544	0.15841	0.12025
N156	0.16323	0.13003	0.07665
C157	0.1465	0.13469	0.08381
N158	0.14842	0.14616	0.10143
N159	0.22469	0.14849	0.10501
N160	0.01029	0.14849	0.10638
Cu161	0.11783	0.15678	0.11773

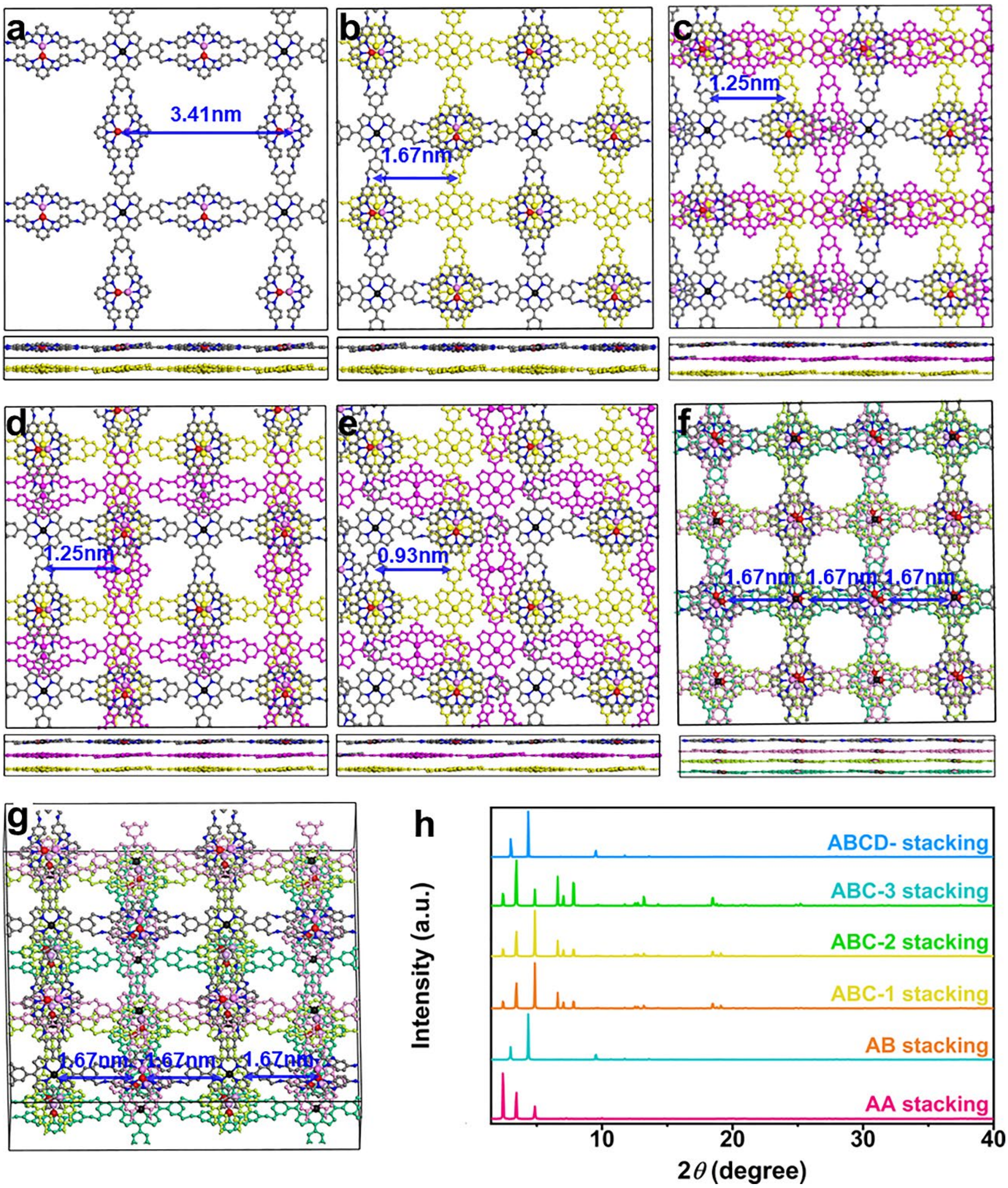

Supplementary Figure 5. Topological representation of ZnPor-RuCuDAC. (a) AA-eclipsed, (b) AB-staggered, and (c–e) slipped ABC-staggered stacking models viewed along the *z*-axis (top) and *x*-axis (bottom). The slipped ABCD-staggered stacking models (f) viewed along the *z*-axis (top) and *x*-axis (bottom) as well as (g) its three dimensions. (h) Simulated PXRD patterns.

Supplementary Table 5. Unit cell parameters and crystal stacking energies of different stacking model for ZnPor-RuCuDAC COF.

Stacking model	AA	AB	ABC-1	ABC-2	ABC-3	ABCD
a = *b* (Å)	38.25	40.57	41.31	41.31	42.57	40.63
c (Å)	5.53	3.81	4.68	4.68	4.03	5.76
α = *β* = *γ* (degree)	90	90	90	90	90	90
Space group	P2/M	P21/M	PMM2	PMM2	PCA21	P21/C
Formula	$C_{256}N_{64}H_{152}Zn_2Ru_4Cu_4$	$C_{256}N_{64}H_{152}Zn_2Ru_4Cu_4$	$C_{384}N_{96}H_{228}Zn_3Ru_6Cu_6$	$C_{384}N_{96}H_{228}Zn_3Ru_6Cu_6$	$C_{384}N_{96}H_{228}Zn_3Ru_6Cu_6$	$C_{512}N_{128}H_{304}Zn_4Ru_8Cu_8$
Total crystal stacking energy per layer (kcal mol^{-1})	57.82	134.53	97.25	97.25	118.79	193.95
Cohesive bulk energy per layer (kcal mol^{-1})	-57.82	-134.53	-97.25	-97.25	-118.79	-163.95

In order to elucidate the structures of these COFs and unit cell parameters, four types of possible 2D structures were generated for ZnPor-RuCuDAC: AA-eclipsed (Supplementary Fig. 4a), AB-staggered (Supplementary Fig. 4b), slipped ABC-staggered (Supplementary Fig. 4c–e) and slipped ABCD-staggered (Supplementary Fig. 4f, g) stacking models. The simulated powder X-ray

diffraction (PXRD) analyses (Supplementary Fig. 4h and Supplementary Tables 2–4) reveal that the AA-eclipsed (A layer: x = 0, y = 0) stacking shows the first intense peak at a low angle of 2.46°, corresponding to the (110) reflection; also, it shows minor peaks at 3.39° and 4.92° for the (202) and (004) reflection planes, respectively. For the AB-staggered (A layer: x = 0, y = 0; B layer: x = 1, y = 1) stacking, the most intense peak corresponding to the (200) reflection plane appears at ~ 4.41°, along with other minor peaks at 3.06° and 9.57° that originate from the (202) and (211) reflection planes, respectively. The slipped ABC-staggered stacking exhibits the same PXRD peaks at 2.46°, 3.39°, 4.81°, 6.62°, 7.08°, 7.89°, 13.25°, and 18.49°, corresponding to the (200), (202), (004), (210), (212), (014), (420), and (031) planes, respectively. Both the ABC-1 (A layer: x = 0, y = 0; B layer: x =1, y = 1; C layer: x =0.5, y = 0) and ABC-2 (A layer: x = 0, y = 0; B layer: x = 1, y = 1; C layer: x = 0, y = 0.5) stacking models show the most intense peak at 4.81°, whereas the slipped ABC-3 (A layer: x = 0, y = 0; B layer: x = 1, y = 1; C layer: x = 0.5, y = 0.5) model shows the strongest peak intensity at 3.39°. The simulated diffraction peaks of ABCD (A layer: x = 0, y = 0; B layer: x = 1, y = 1; C layer: x = 1, y = 0; D layer: x = 0, y = 1) model are similar to that of AB stacking. However, it should be noted that the spatial distance of the adjacent Zn, Cu, and Ru are ca. 2.36 (along the diagonal) or 3.34 nm (along the x/y axis) according to the AB-staggered stacking models, while the distance of the adjacent Zn, Cu, and Ru are ca. 1.67 (along x/y axis) or 2.36 nm (along the diagonal) with ABCD stacking.

Further, the density functional tight binding method with Lennard–Jones function was utilized to evaluate crystal stacking energies of ZnPor-RuCuDAC quantitatively (Supplementary Table 5). Evidently, the AA-eclipsed, AB-staggered, slipped ABC-1, slipped ABC-2, slipped ABC-3 and slipped ABCD stacking exhibit total per-layer crystal stacking energies of 57.82, 134.53, 97.25, 97.25, 118.79 and 193.95 kcal mol^{-1}, respectively, indicating that ABCD-staggered stacking is more favorable than the other five isomeric structures. The experimental PXRD profiles of ZnPor-RuCuDAC, ZnPor-Ru_2DAC, and ZnPor-Cu_2DAC exhibit three main diffraction peaks (Fig. 1b–d, experimental profiles in black, Pawley-refined profiles in red, predicted profiles in magenta, and differences in blue) at 3.06°, 4.42°, and 9.57°, which are consistent with those obtained in the simulated diffraction pattern of AB- and ABCD-staggered stacking model. This result suggests that

these diatomic COFs adopt an AB- or ABCD-stacked structure. Moreover, according to the nonlocal density functional theory, these COFs with AA-eclipsed, AB-staggered, slipped ABC-1, slipped ABC-2, slipped ABC-3 and slipped ABCD stacking models show pore size distributions (Supplementary Fig. 4b–f) centered at 3.41, 1.67, 1.25, 1.25, 0.93, and 1.67 nm, respectively. However, the parameters of individual building compartments from AC-ADF-STEM (Fig. 1g) are measured with a = b = 1.67 ± 0.1 nm (α = 90 ± 0.3°) in ZnPor-RuCuDAC COF. The 3D electron diffraction tomography of DPC images (Fig. 1h–j) reveal the spatial distances among adjacent Zn, Cu, and Ru are ca. 1.67 nm, which is equably atomic cross-distribution on the ZnPor-RuCuDAC substrate. These results confirm that the COF photocatalysts possibly present ABCD-staggered stacking structure rather than the simple AB-staggered configuration, which is evidenced by the negligible difference obtained in the Pawley refinement results.

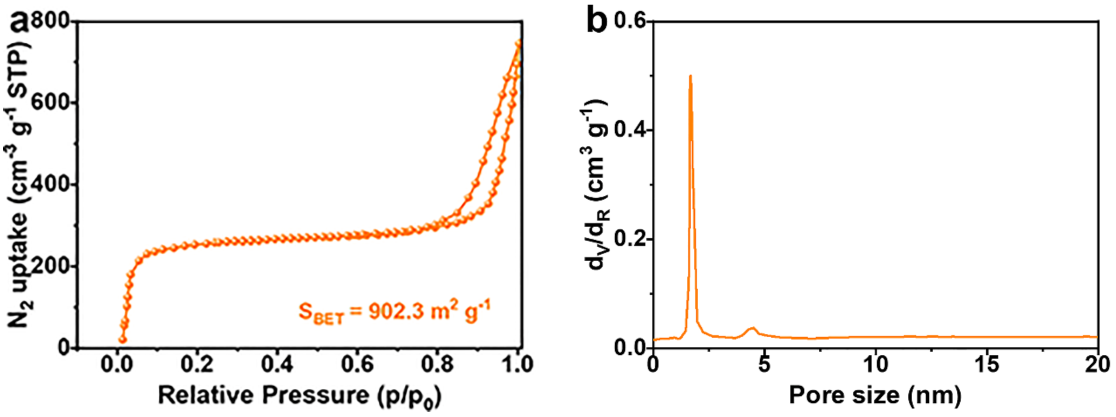

Supplementary Figure 6. (a) N_2 adsorption-desorption isotherms and (b) pore size distribution of ZnPor-RuCuDAC at 77 K.

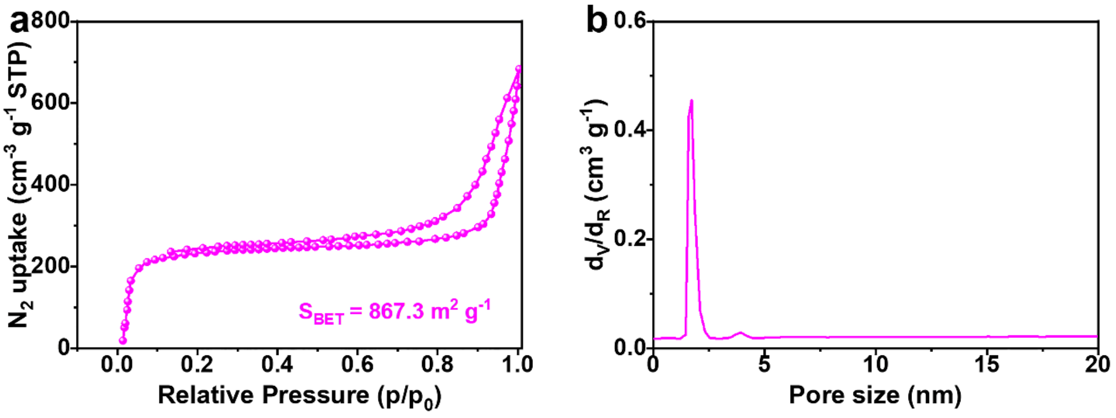

Supplementary Figure 7. (a) N_2 adsorption-desorption isotherms and (b) pore size distribution of ZnPor-Ru_2DAC at 77 K.

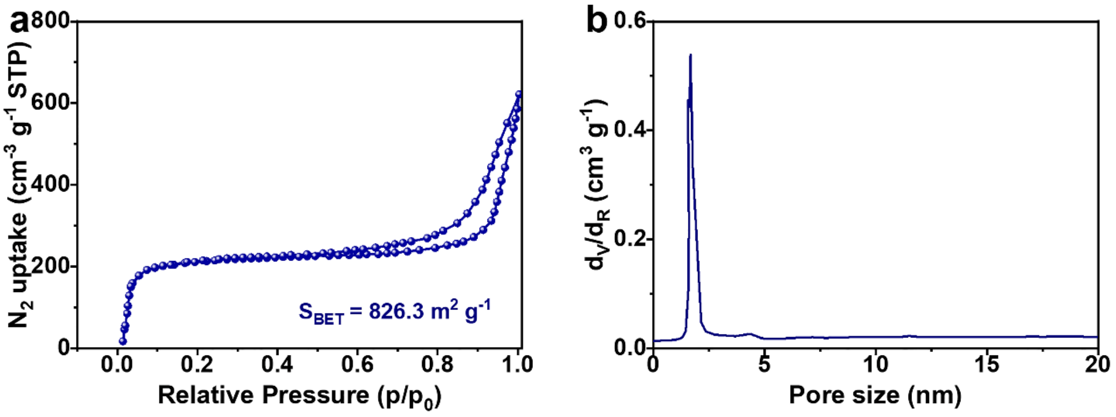

Supplementary Figure 8. (a) N_2 adsorption-desorption isotherms and (b) pore size distribution of ZnPor-Cu_2DAC at 77 K.

The Brunauer–Emmett–Teller (BET) surface areas were estimated as 902.3 (ZnPor-RuCuDAC COF), 867.3 (ZnPor-Ru_2DAC COF), and 826.3 (ZnPor-Cu_2DAC COF) m^2 g^{-1}, with pore volumes of 0.36, 0.31, and 0.25 cm^3 g^{-1}, respectively. By the desorption branches, the Barret-Joyner-Halenda (BJH) pore size distribution plots are obtained. ZnPor-RuCuDAC display two peaks centered at 1.66 and 4.32 nm, ZnPor-Ru_2DAC show two major peaks at 1.68 and 4.46 nm, and ZnPor-Cu_2DAC exhibit two peaks centered at 1.65 and 4.17 nm. The pore size of lower region is the capillary condensation of N_2 molecules among the square-like pores within the COFs, while the pore size of the high one can be ascribed to N_2 capillary condensation at the interlayer of COF nanosheet.

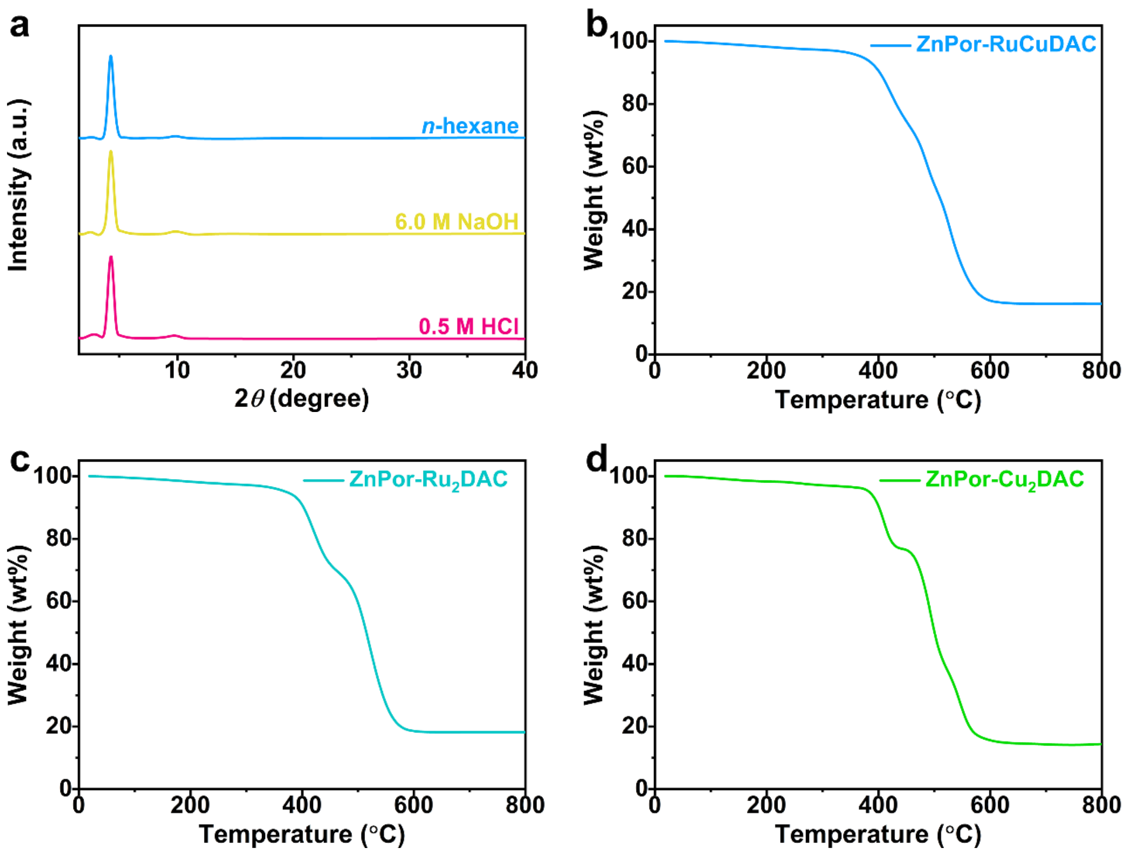

Supplementary Figure 9. Stability analyses of ZnPor-RuCuDAC. (a) PXRD pattern of ZnPor-RuCuDAC before and after treated in different solvents for 40 h. In a typical experiment, 10 mg of ZnCu-COF was immersed in the solvents (n-hexane, 0.5 M HCl, 6.0 M NaOH) at room temperature. Thermogravimetric curves of (b) ZnPor-RuCuDAC, (c) ZnPor-Ru_2DAC, and (d) ZnPor-Cu_2DAC in the air.

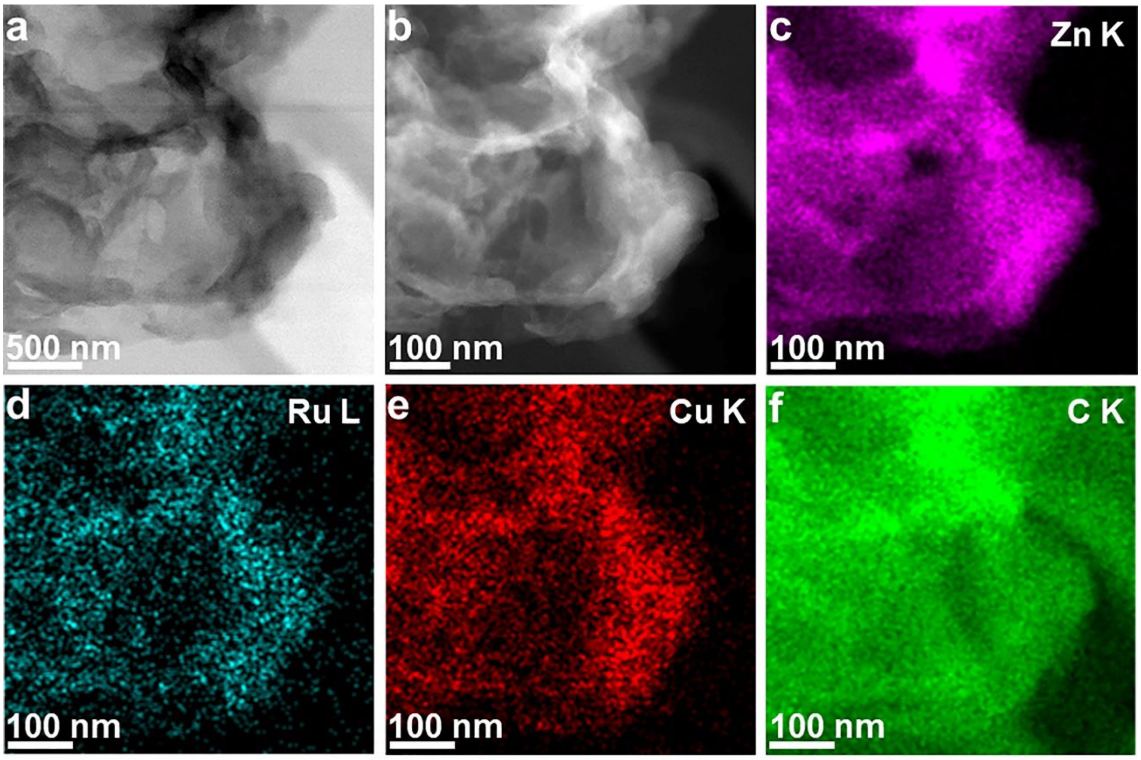

Supplementary Figure 10. Microstructural visualization of the ZnPor-RuCuDAC COF. (a,b) High-resolution AC-TEM images and (c–f) the corresponding element maps.

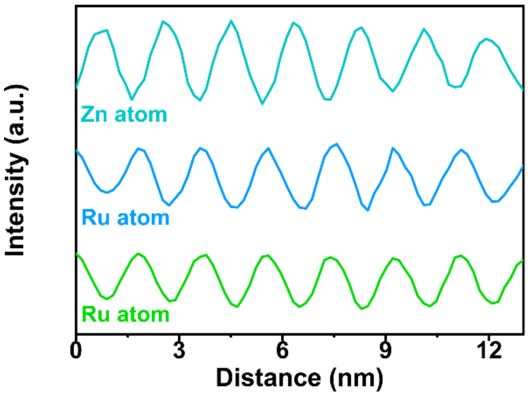

Supplementary Figure 11. The relative Zn, Ru, and Cu atom spatial location in ZnPor-RuCuDAC-COF backbone, obtained from 3D electron diffraction tomography of DPC images.

Supplementary Table 6. Edge energy of Zn, Ru, and Cu K-edge XANES spectra for different samples.

Sample	Zn (eV)	Sample	Ru (eV)	Sample	Cu (eV)
Zn foil	9645.8	Ru foil	22102.6	Cu foil	8948.1
Zn Pc	9666.9	$RuCl_3$	22157.0	CuPc	8970.0
ZnPor	9663.5	RuN_3	22140.6	CuN_3	8966.7
ZnPor-RuCuDAC	9668.8	ZnPor-RuCuDAC	22136.7	ZnPor-RuCuDAC	8962.7
ZnPor-Ru_2DAC	9671.5	ZnPor-Ru_2DAC	22132.3	ZnPor-Cu_2DAC	8964.3
ZnPor-Cu_2DAC	9667.4	-	-	-	-

Supplementary Table 7. EXAFS curve-fitting results for the structural parameters around Zn atom of various samples.

Sample	Shell	N	*R* (Å)	σ^2 (× 10^{-3} Å)	ΔE_0 (eV)	*R* factor
Zn foil	Zn–Zn	12	2.66	1.5	5.8	0.083
Zn Pc	Zn–N	4.1	1.45	1.7	2.5	0.076
ZnPor	Zn–N	4.0	1.44	2.1	3.6	0.009
ZnPor-	Zn–N	4.1	1.43	2.7	2.7	0.013
ZnPor-Ru_2DAC	Zn–N	3.9	1.43	3.1	3.1	0.019
ZnPor-Cu_2DAC	Zn–N	4.0	1.43	4.3	1.9	0.027

Shell: Coordination atom; N: Coordination number; *R*: Bond length; σ^2: Debye-Waller factor; ΔE_0: Inner potential correction; *R* factor: Goodness of fit. The fit was optimized in *R* space with a **k**-weight of 3. S_0^2 of Co-N path was set to 0.96 according to the fitting for experimental EXAFS of ZnPc.

Supplementary Table 8. EXAFS curve-fitting results for the structural parameters around Ru atom of various samples.

Sample	Shell	N	*R* (Å)	σ^2 (× 10^{-3} Å)	ΔE_0 (eV)	*R* factor
Ru foil	Ru–Ru	12.0	2.76	4.5	7.3	0.007
$RuCl_3$	Ru–Cl	6.1	1.98	3.8	6.8	0.138
RuN_3	Ru–N	3.1	1.43	2.2	5.4	0.009
	Ru–Cl	3.0	1.99	2.5	5.4	0.009
ZnPor-	Ru–N	3.1	1.43	3.2	3.8	0.037
	Ru–Cu	1.0	2.70	3.4	3.8	0.037
ZnPor-Ru_2DAC	Ru–N	6.0	1.43	2.9	4.6	0.018
	Ru–Ru	1.0	2.68	3.1	4.6	0.018

Shell: Coordination atom; N: Coordination number; *R*: Bond length; σ^2: Debye-Waller factor; ΔE_0: Inner potential correction; *R* factor: Goodness of fit. The fit was optimized in *R* space with a **k**-weight of 3. S_0^2 of Ru-N path was set to 0.92 according to the fitting for experimental EXAFS of $RuCl_3$.

Supplementary Table 9. EXAFS curve-fitting results for the structural parameters around Cu atom of various samples.

Sample	Shell	N	*R* (Å)	σ^2 (× 10^{-3} Å)	ΔE_0 (eV)	*R* factor
Cu foil	Cu–Cu	12.0	2.68	3.2	2.4	0.017
CuPc	Cu–Cl	4.0	1.48	3.8	3.5	0.072
CuN_3	Cu–N	3.0	1.47	1.7	3.2	0.006
	Cu–Cl	3.0	2.07	2.1	3.2	0.006
ZnPor-	Cu–N	3.1	1.47	1.6	3.6	0.016
	Ru–Cu	1	2.61	1.8	3.6	0.016
ZnPor-Cu_2DAC	Cu–N	5.9	1.47	2.6	1.9	0.027
	Cu–Cu	1.0	2.59	2.9	1.9	0.027

Shell: Coordination atom; N: Coordination number; *R*: Bond length; σ^2: Debye-Waller factor; ΔE_0: Inner potential correction; *R* factor: Goodness of fit. The fit was optimized in *R* space with a **k**-weight of 3. S_0^2 of Cu-N path was set to 0.95 according to the fitting for experimental EXAFS of CuPc.

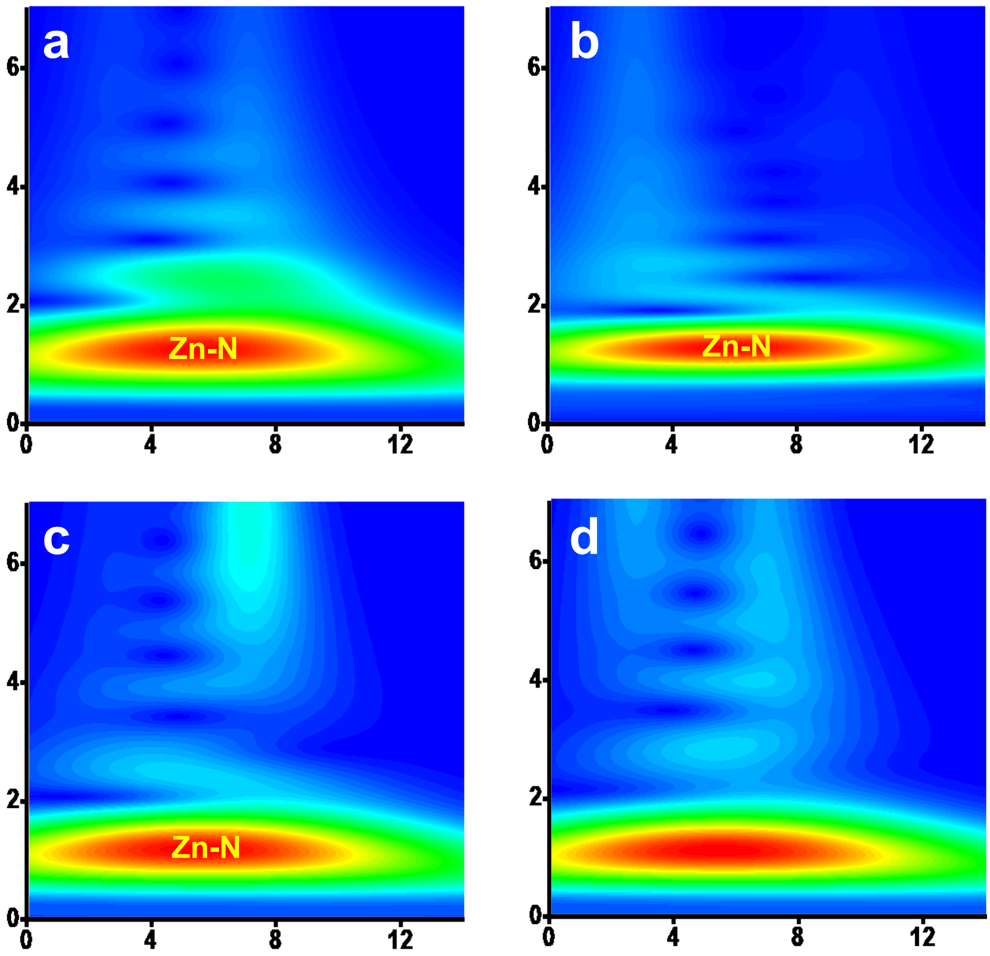

Supplementary Figure 12. Wavelet transforms of Zn **k**^{3}-weighted EXAFS for ZnPor (a), ZnPor-RuCuDAC (b), ZnPor-Ru_2DAC (c), and ZnPor-Cu_2DAC (d).

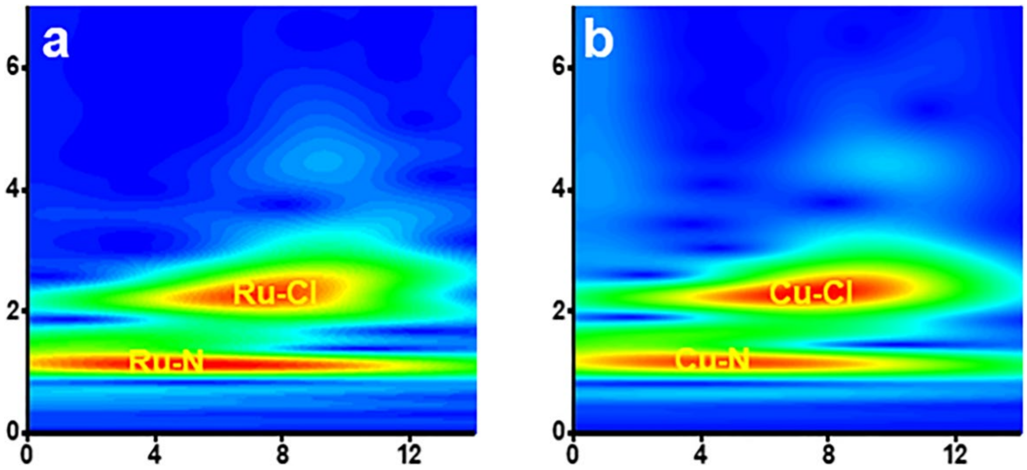

Supplementary Figure 13. Wavelet transforms of Ru **k**^{3}-weighted EXAFS for RuN_3 (a) and Cu **k**^{3}-weighted EXAFS for CuN_3 (b).

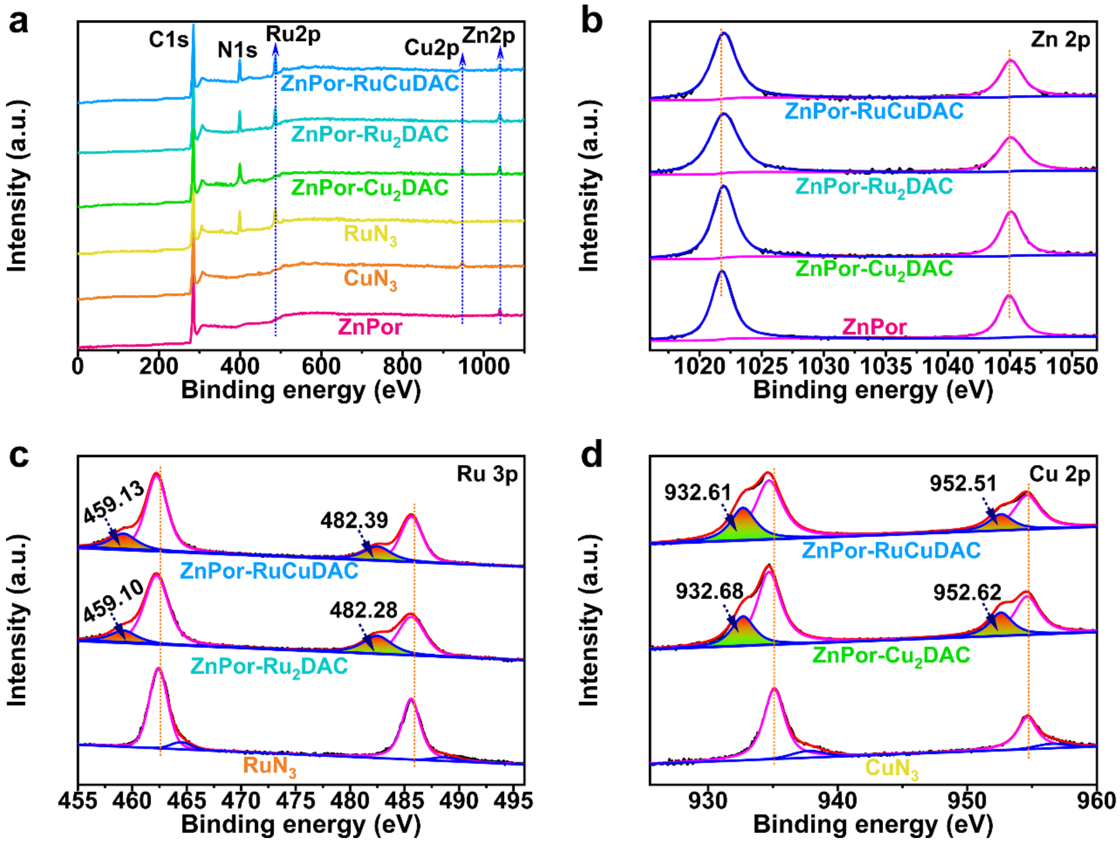

Supplementary Figure 14. XPS analyses of various COFs. Survey (a), high-resolution Zn2*p* (b), Ru3*p* (c), and Cu2*p* (d) XPS spectra of ZnPor-RuCuDAC, ZnPor-Ru_2DAC, ZnPor-Cu_2DAC, and their monomers, respectively.

The survey XPS (Supplementary Fig. 13a) indicates that Zn, Ru, Cu, C, and N coexist in the precursors and/or these COFs. The high-resolution Zn2*p* XPS spectra (Supplementary Fig. 13b) show that ZnPor has Zn(II) $2p_{3/2}/2p_{1/2}$ binding energy (BE) peaks of 1021.81/1044.95 for Zn–N bond, which shows a positive shift for ZnPor-RuCuDAC (~0.18 eV), ZnPor-Ru_2DAC (~0.22 eV) and ZnPor-Cu_2DAC (~0.23 eV), ascribable to a decreased electron density of the ZnPor cores. The high-resolution Ru3*p* XPS spectrum (Supplementary Fig. 13c) of RuN_3 exhibits two BE peaks at 462.57/485.83 and 464.43/488.93 eV for Ru–N and Ru–Cl bond, belonging to the Ru(II) species, which exhibit a positive shift for ZnPor-RuCuDAC and ZnPor-Ru_2DAC for an increased electron density of RuN_3 cores. However, as compared to RuN_3 monomer, ZnPor-RuCuDAC, and ZnPor-Ru_2DAC show new binding energy peaks at 459.13/482.39 and 459.10/482.28 eV, respectively,

which can be ascribable to the formation of Ru–Cu (ZnPor-RuCuDAC) or Ru–Ru (ZnPor-Ru_2DAC) diatomic pairs.

Similarly, CuN_3 monomer present BE peaks of Cu(II) $2p_{3/2}/2p_{1/2}$ for Cu–N and Ru–Cl bond (Supplementary Fig. 13d), which show a positive shift for ZnPor-RuCuDAC and ZnPor-Cu_2DAC for an increased electron density of CuN_3 cores. Contrastingly, as compared to CuN_3 monomer, ZnPor-RuCuDAC and ZnPor-Cu_2DAC show new BE peaks at 932.61/952.51 and 932.68/952.62 eV, respectively, which can be ascribable to the formation of Ru–Cu (ZnPor-RuCuDAC) or Cu–Cu (ZnPor-Cu_2DAC) diatomic pairs. The above-fitted XPS spectra further indicate that compared to the single-atom Zn distribution, the Ru–Cu, Ru–Ru or Cu–Cu diatomic pairs are formed on the COF skeletons. Furthermore, the decreased electron density of ZnPor cores and increased one of RuN_3/CuN_3 can be ascribable to the intensive metal-to-metal charge transfer behaviors from ZnBPP to RuN_3/CuN_3 cores, demonstrating that energy transfer and electron diffusion occurred among the hetero-trimetallic cores in the extended two-dimensional networks of these COFs.

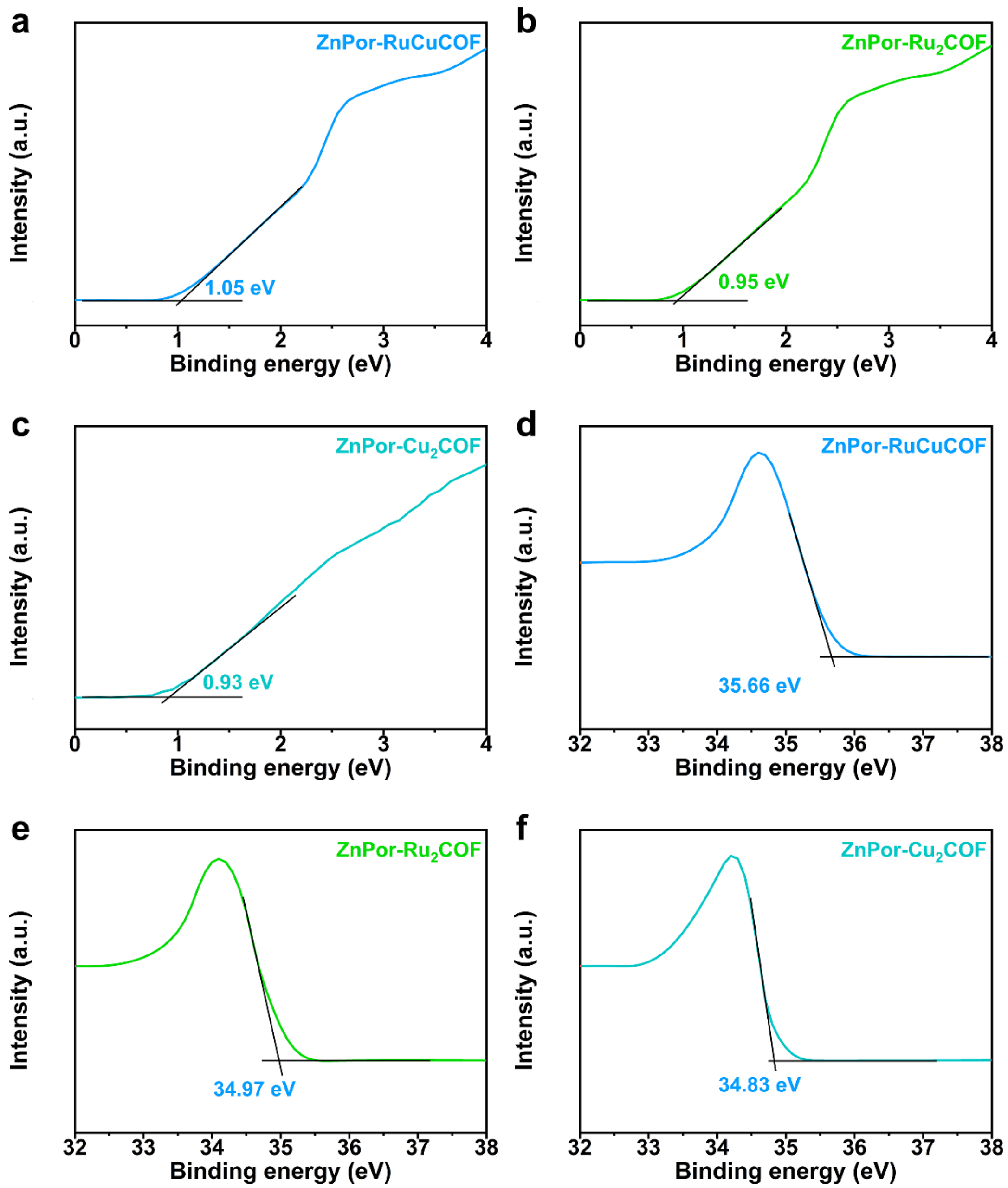

Supplementary Figure 15. (a–c) Valence band spectra and (d–f) secondary electron cutoff for ZnPor-RuCuDAC, ZnPor-Ru_2DAC, and ZnPor-Cu_2DAC, respectively, measured by synchrotron-radiation photoemission spectroscopy.

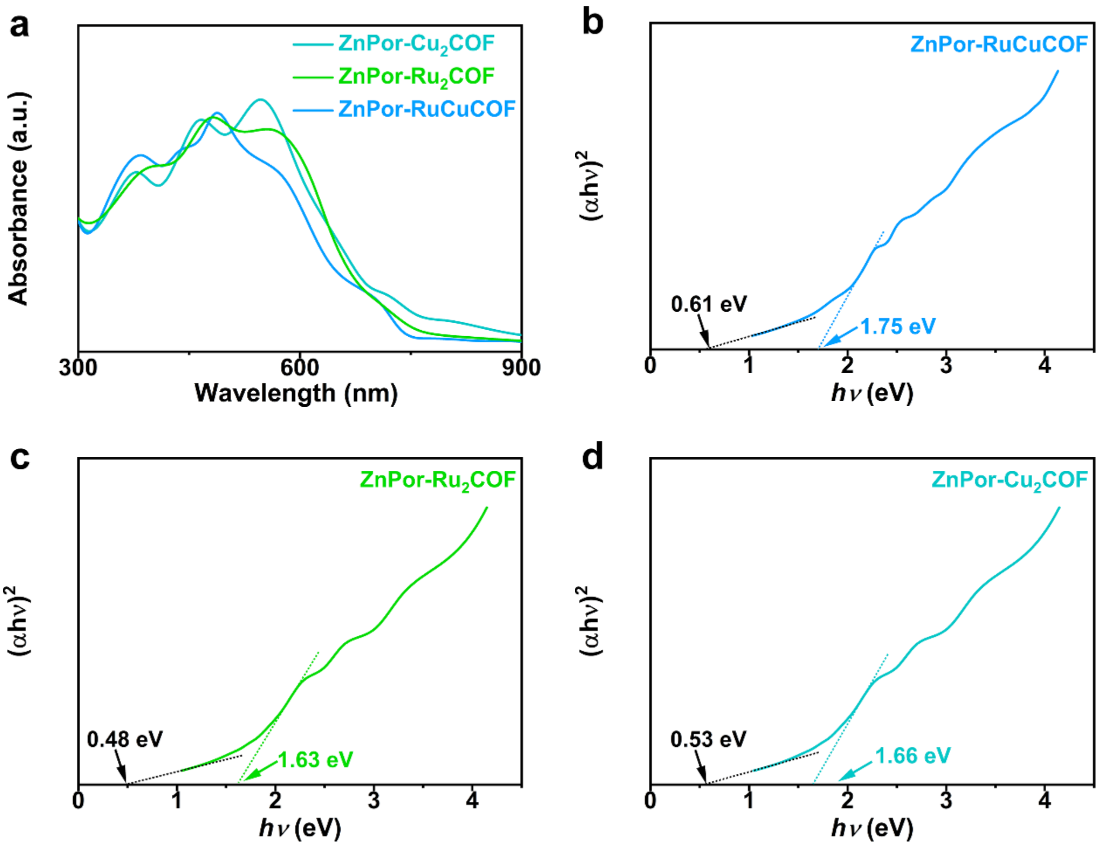

Supplementary Figure 16. (a) Ultraviolet-visible diffuse reflection spectra of the COFs. Obtained bandgaps by plotting $(\alpha h\nu)^2$ vs. hν for the (b) ZnPor-RuCuDAC, (c) ZnPor-Ru_2DAC, and (d) ZnPor-Cu_2DAC, respectively. From Kubelka–Munk method, a broad band gap can be calculated for ZnPor-RuCuDAC (1.75 eV), ZnPor-Ru_2DAC (1.63 eV), and ZnPor-Cu_2DAC (1.66 eV), respectively; A narrow band gap can also be obtained for ZnPor-RuCuDAC (0.61 eV), ZnPor-Ru_2DAC (0.48 eV) and ZnPor-Cu_2DAC (0.53 eV). Since these synthesized metalloporphyrin COFs have a claret color with ca. 720 nm absorption edge, it can be concluded that they are an intrinsic semiconductor with broad bandgap.

Supplementary Table 10. Valence band maxima (VBM) and conduction band minima (CBM) of the ZnPor-RuCuDAC, ZnPor-Ru_2DAC, and ZnPor-Cu_2DAC.

Band	ZnPor-RuCuDAC (eV)	ZnPor-Ru_2DAC (eV)	ZnPor-Cu_2DAC (eV)
VBM	1.05	0.95	0.93
CBM	–0.70	–0.68	–0.73

Supplementary Table 11. Reduction potentials of the reactions involved in CO_2 reduction.

Reaction	E^o (eV vs. NHE)
CO_2 (g) + 2e$^-$ + 2H$^+$ → CO (g)	–0.53
CO_2 (g) + 2e$^-$ + 2 H$^+$ → HCOOH	–0.61
CO_2 (g) + 8e$^-$ + 8H$^+$ → CH_3COOH	–0.18
H_2O (g) + 2e$^-$ +2 H$^+$ → H_2 (g)	0

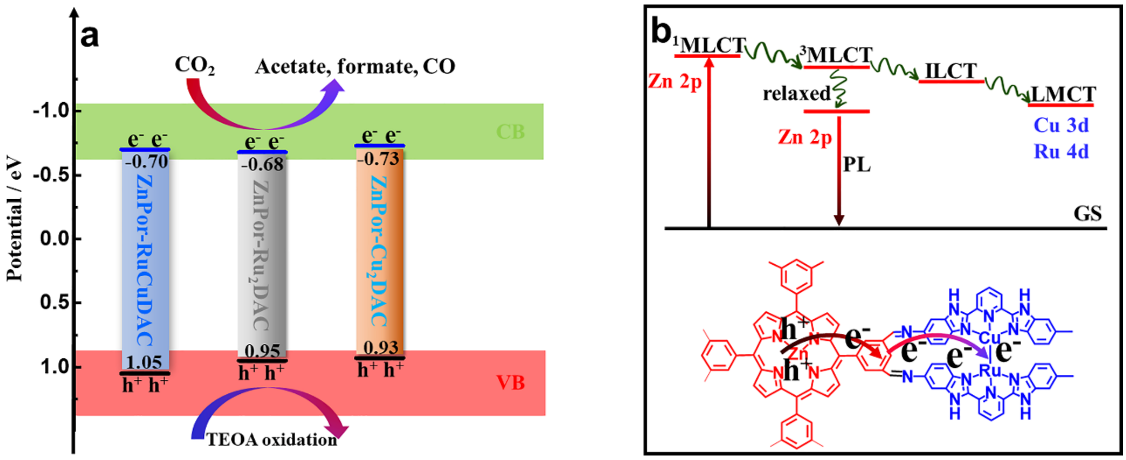

Supplementary Figure 17. Schematic diagram of (a) energy band structures and (b) simplified model for electron diffusion within these PMD-like diatomic COFs. (GS = ground state, PL = photoluminescence, MLCT = metal-to-ligand charge transfer, ILCT = intra-ligand charge transfer, and LMCT = ligand-to-metal charge transfer)

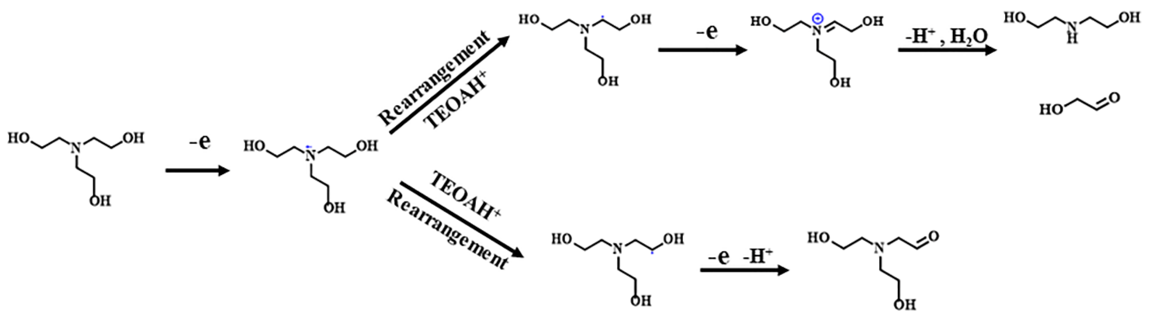

Supplementary Figure 18. Mechanism of triethanolamine degradation as a sacrificial reagent in the photocatalytic system. Once interacting with the excited ZnPor photosensitive centers, a positively charged aminyl radical is formed. The deprotonation of the aminyl radical by TEOA itself leads to a rearrangement into a carbon-centered radical displaying a significant reductive power. The iminium species further degrades into hydroxy ethanal and secondary amine by hydrolysis of the iminium in aqueous media.

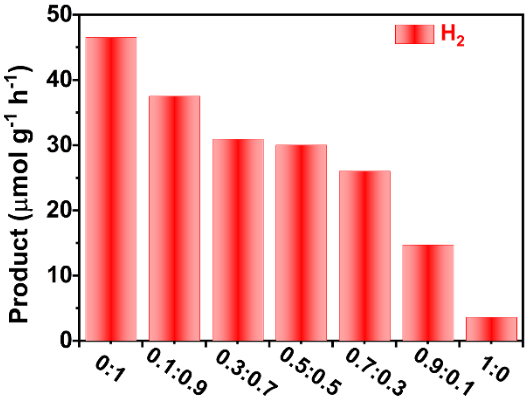

Supplementary Figure 19. The evolution rate of H_2 on diatomic COFs with different Ru:Cu molar ratios.

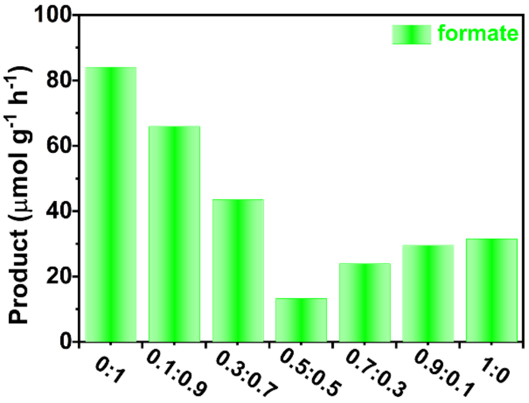

Supplementary Figure 20. The evolution rate of formate on diatomic COFs with different Ru:Cu molar ratios.

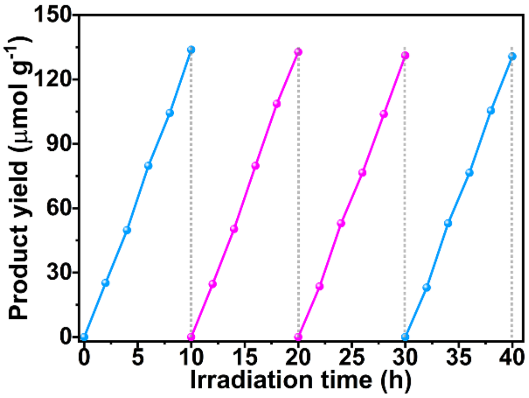

Supplementary Figure 21. The rate of formate produced in four consecutive runs (10 hours for each circle) of photocatalytic CO_2 reaction over ZnPor-RuCuDAC.

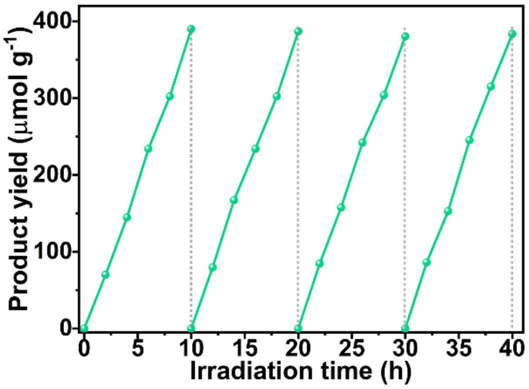

Supplementary Figure 22. The rate of CO produced in four consecutive runs (10 hours for each circle) of photocatalytic CO_2 reaction over ZnPor-RuCuDAC.

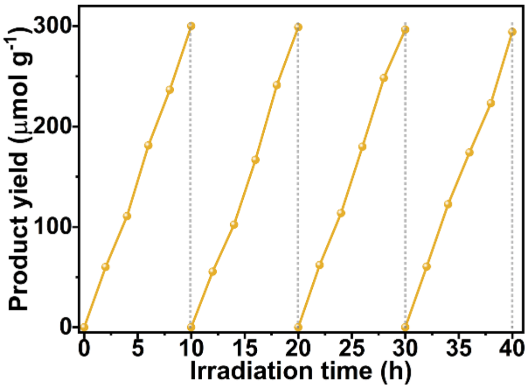

Supplementary Figure 23. The rate of H_2 produced in four consecutive runs (10 hours for each circle) of photocatalytic CO_2 reaction over ZnPor-RuCuDAC.

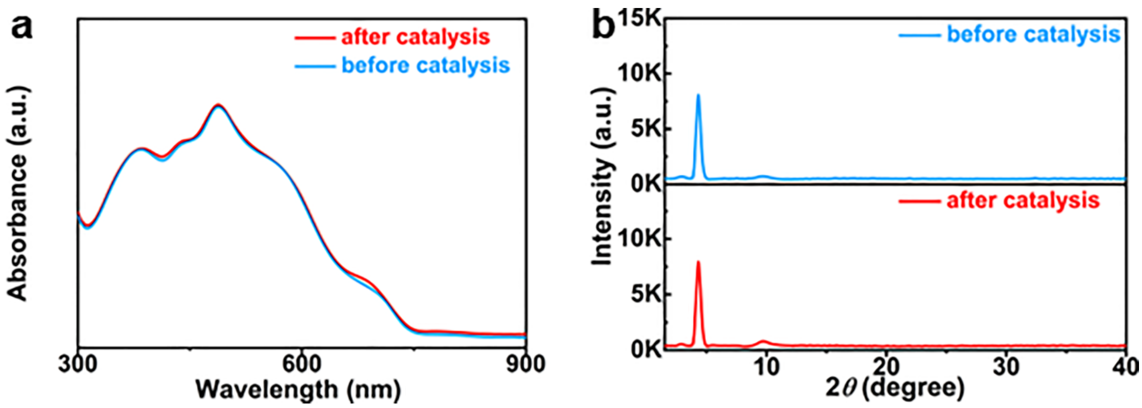

Supplementary Figure 24. (a) DRS and (b) PXRD patterns of ZnPor-RuCuDAC before and after 40 h photoreaction.

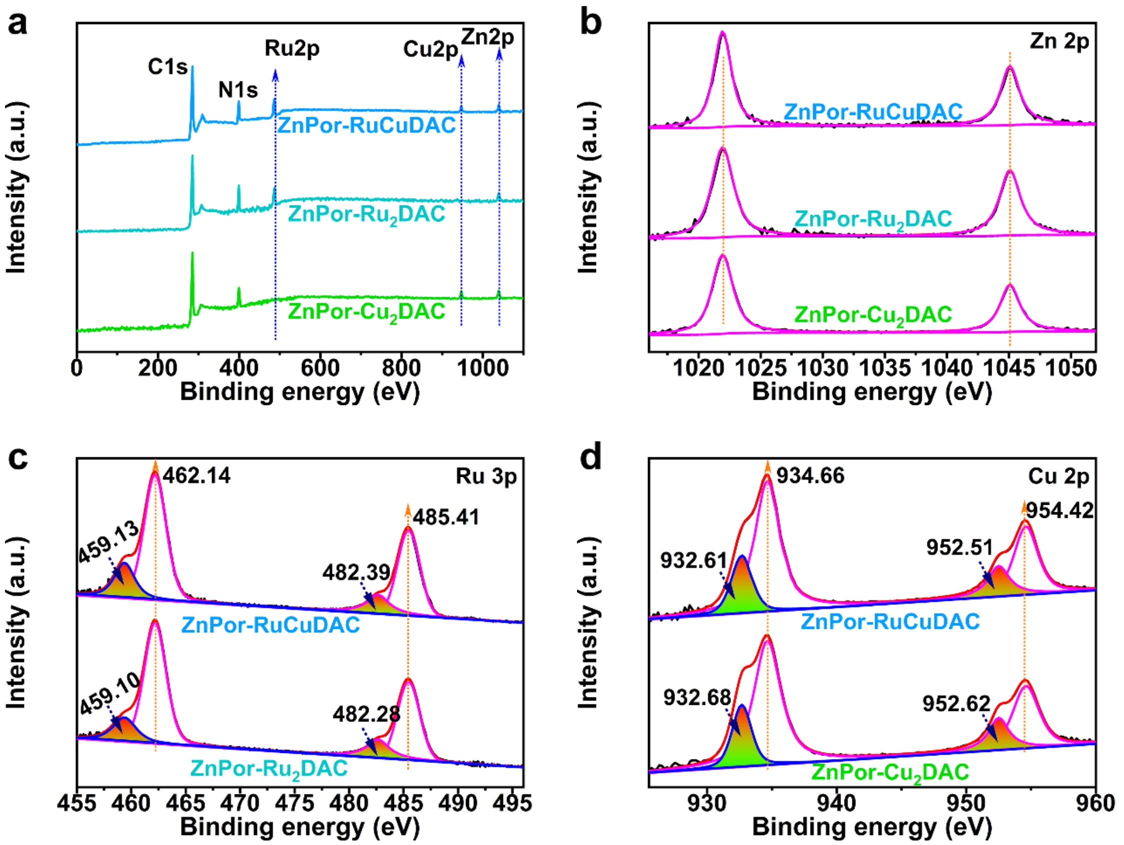

Supplementary Figure 25. Survey (a), high-resolution Zn2*p* (b), Ru3*p* (c), and Cu2*p* (d) XPS spectra of ZnPor-RuCuDAC, ZnPor-Ru_2DAC, ZnPor-Cu_2DAC after 40 h photoreaction, respectively. The survey XPS spectrum of the recovered COFs product after photocatalytic reaction indicates that Zn, Ru, Cu, C, and N still coexist in the COFs' skeleton. The high-resolution recovered Zn2*p*, Cu2*p,* and Ru 3*p* XPS spectra have Zn(II), Ru(II) and Cu(II) binding energy peaks, respectively, which are the same as that of diatomic COFs without photoreaction. These indicate that the element composition and oxidation states of ZnPor-RuCuDAC, ZnPor-Ru_2DAC, and ZnPor-Cu_2DAC are unchanged during the long-term photoreaction process.

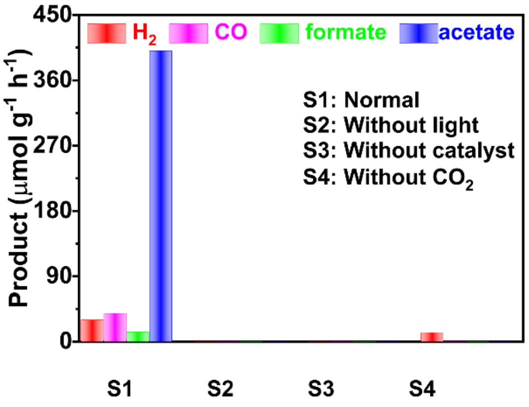

Supplementary Figure 26. Control experiments under different reaction conditions for CO_2 reduction over the ZnPor-RuCuDAC. For each control experiment, other conditions remained the same compared to the photoreduction of the ZnPor-RuCuDAC.

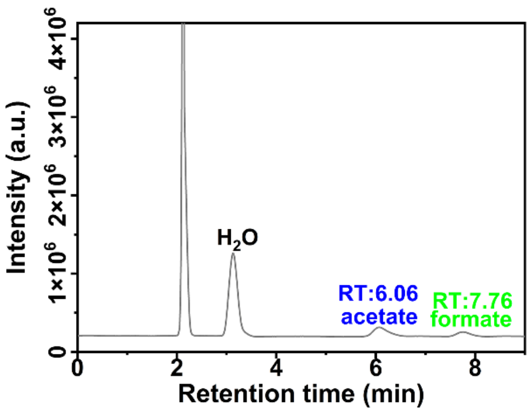

Supplementary Figure 27. Total ion chromatogram of liquid product for $^{13}CO_2$ photoreduction with HP-FFAP column.

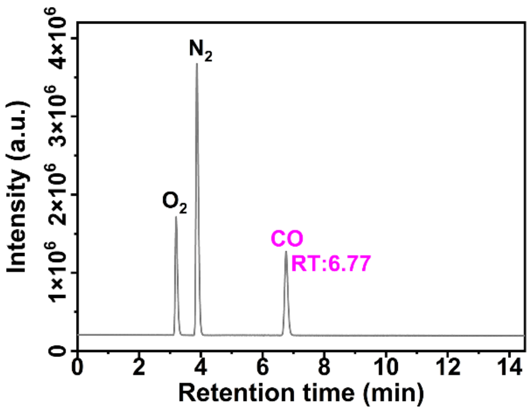

Supplementary Figure 28. Total ion chromatogram of gaseous product for $^{13}CO_2$ photoreduction with HP-Molesieve column.

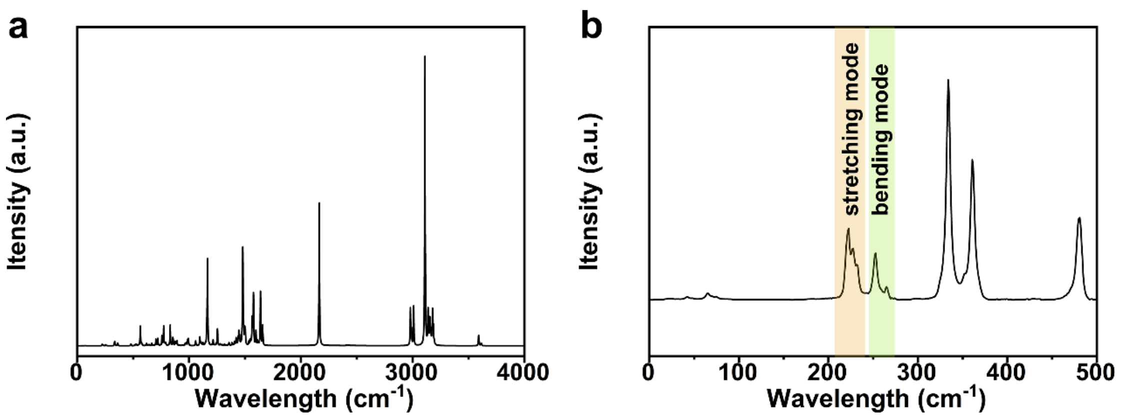

Supplementary Figure 29. (a) Simulated Raman spectra of ZnPor-RuCuDAC and (b) the zoomed Raman peaks in the region of 0–500 cm^{-1} of Supplementary Figure 29(a). According to vibration-frequency analysis of molecular structure, the peak at ~226 and 252 cm^{-1} can be ascribable to the Ru–Cu stretching and bending vibration, respectively.

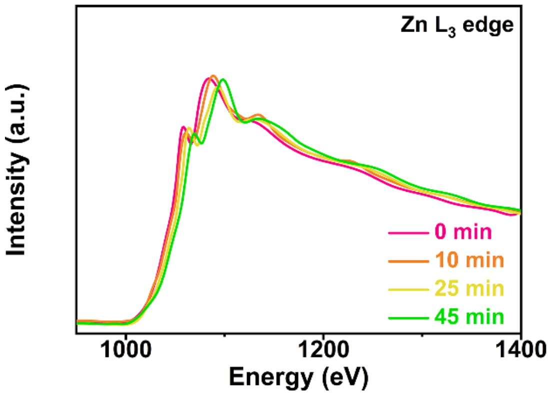

Supplementary Figure 30. XAS Zn L_3-edge spectra of ZnPor-RuCuDAC under different illumination times.

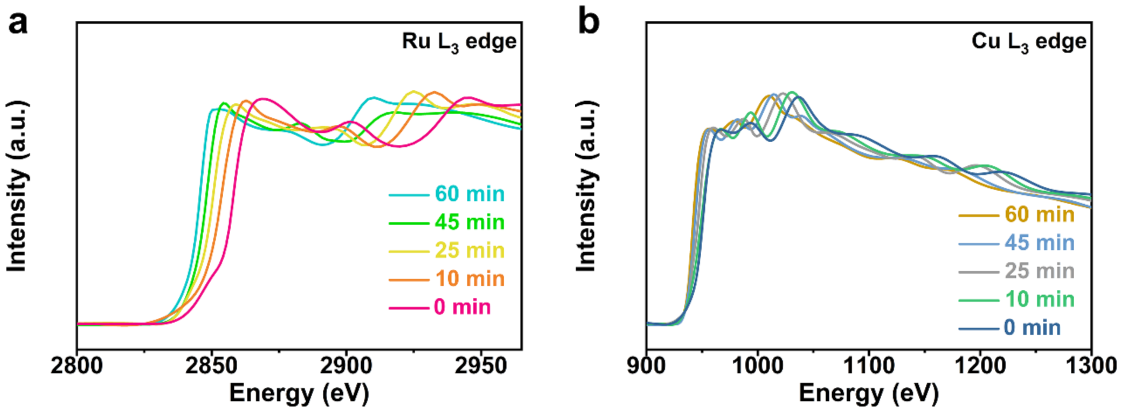

Supplementary Figure 31. XAS (a) Ru L_3-edge and (b) Cu L_3-edge spectra of ZnPor-RuCuDAC under different illumination times.

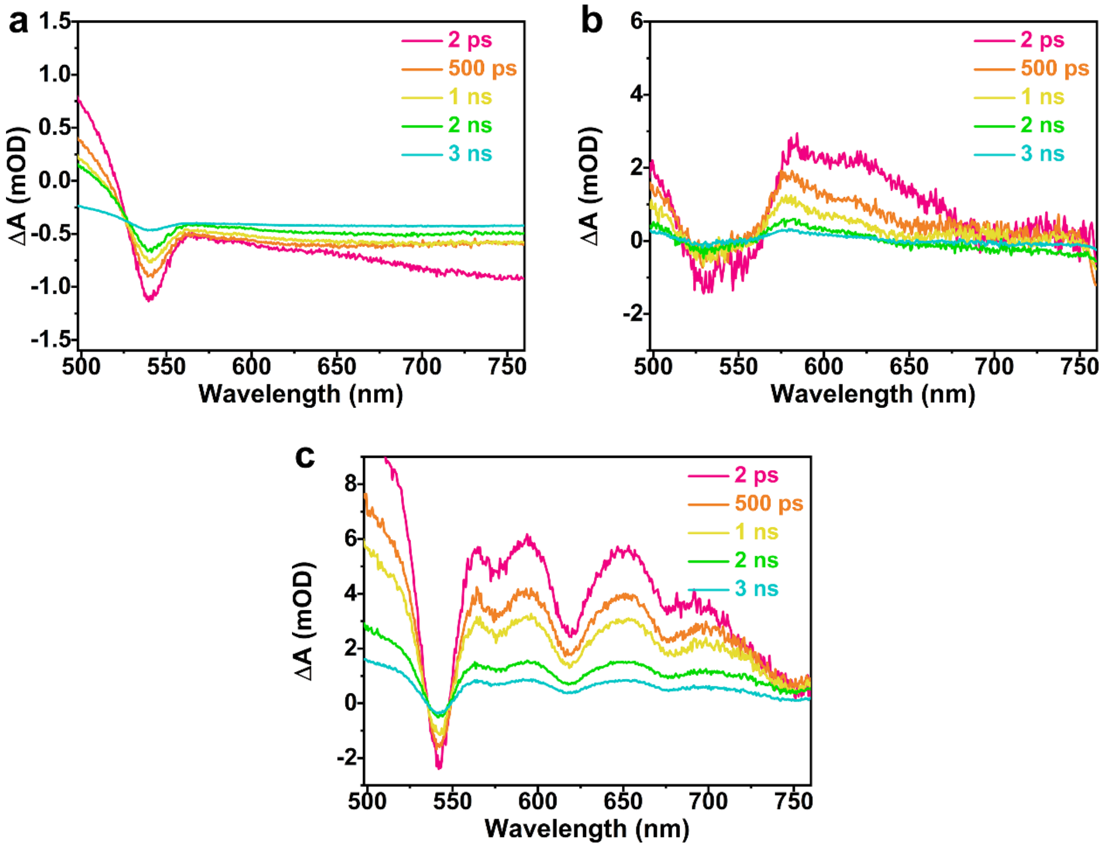

Supplementary Figure 32. Femtosecond transient spectra of (a) ZnPor-N_3 COF, (b) H_2Por-RuCuDAC, and (c) ZnPor-RuCuDAC obtained at 400 nm excitation at different pump-probe delay times (2 ps, 500 ps, 1 ns, 2 ns, and 3 ns).

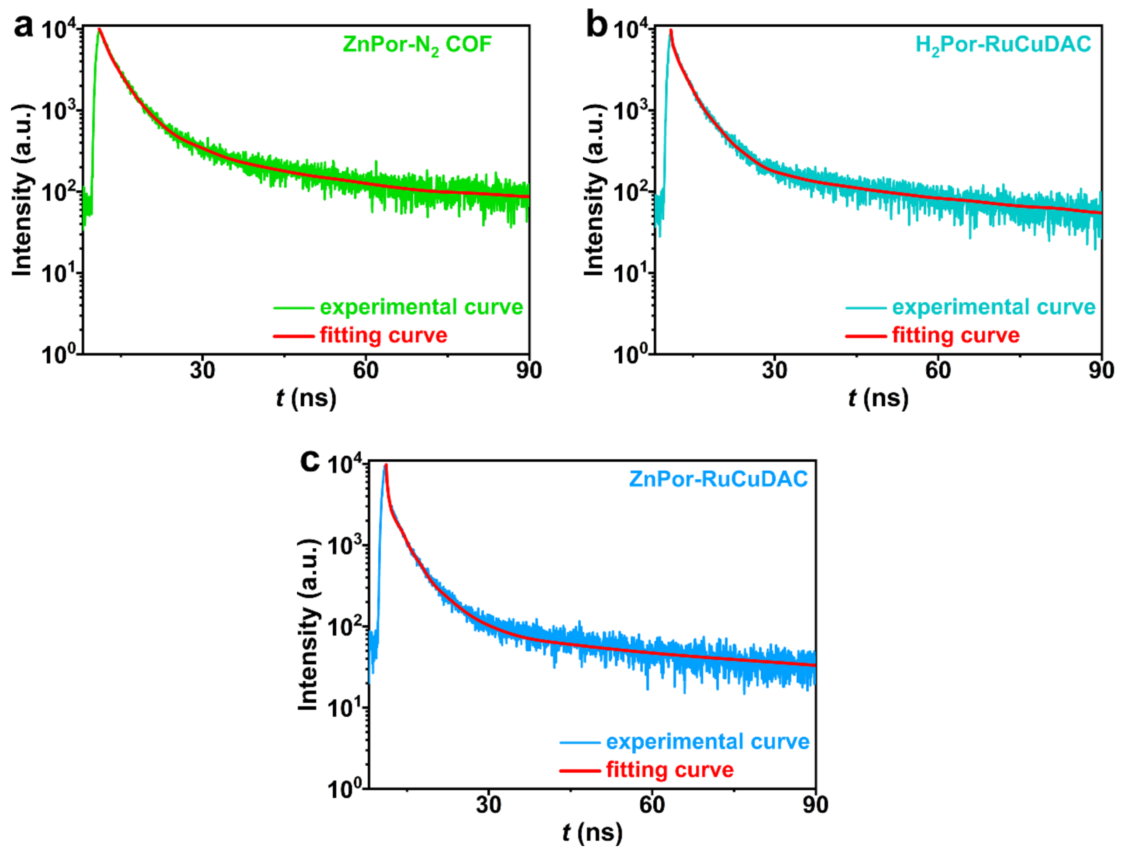

Supplementary Figure 33. TRPL spectra of (a) ZnPor-N$_3$ COF, (b) H_2Por-RuCuDAC, and (c) ZnPor-RuCuDAC excited and detected by 400 and 561 nm, respectively.

Supplementary Table 12. Comparison of the fluorescence decay data of ZnPor-N$_3$ COF, H_2Por-RuCuDAC, and ZnPor-RuCuDAC.

sample	ZnPor-N$_3$ COF	H_2Por-RuCuDAC	ZnPor-RuCuDAC
τ_1 (ns)	0.83	0.72	0.63
τ_2 (ns)	6.73	5.86	3.39
τ_{ave} (ns)	3.15	2.56	1.53

For comparison, metal-deficient COF counterparts of ZnPor-N$_3$ COF (Ru- and Cu-free) and H_2Por-RuCuDAC (Zn-free) were synthesized for reaching a comprehensive understanding of the charge-transfer mechanism between the ZnPor unit and dual-atom active sites, and the corresponding picosecond transient absorption spectroscopy (TA) was performed (Supplementary Fig. 32). After being excited by a pump pulse with a wavelength of 400 nm, the TA spectra of ZnPor-N$_3$ COF

showed a pronounced negative peak at ca. 542 nm, which is assigned to ground-state bleach (GSB) and reflects the excited state relaxation. Except for the GSB peak, an extra positive absorption band at ca. 553–684 nm was observed in the TA spectra of H_2Por-RuCuDAC COF at delay time, but it was not detected in the spectra of ZnPor-N_3 COF. Furthermore, ZnPor-RuCuDAC exhibits a much broader and more intensive absorption band at 552–736 nm. This finding demonstrates that the fluctuant TA of H_2Por-RuCuDAC and ZnPor-RuCuDAC is attributed to the charge transfer between ZnPor (Por) cores and Ru–Cu diatomic sites according to the energy transfer and electron diffusion procedure of MLCT, ILCT, and LMCT. This conjecture can be further confirmed by picosecond TRPL spectra (Supplementary Fig. 33 and Supplementary Table 12) of these COFs, where the time decays are fitted with a double exponential function ($\Delta A(t) = \Delta A_0 + A_1e^{-t/\tau1} + A_2e^{-t/\tau2}$), resulting in one component with a shorter lifetime (τ_1, contributing radiative fluorescence quenching) and another component with a longer lifetime (τ_2, reflecting nonradiative recombination). As seen, the ZnPor-RuCuDAC showed the fastest decay lifetime than ZnPor-N_3 COF and H_2Por-RuCuDAC, especially for τ_2 lifetime with 49.6% percentage decay. The average fluorescence lifetimes (τ_{ave}) were calculated to be 3.15 (ZnPor-N_3 COF), 2.56 (H_2Por-RuCuDAC), and 1.53 (ZnPor-RuCuDAC) ns, respectively, implying more fluent photogenerated charge separation over ZnPor-RuCuDAC.

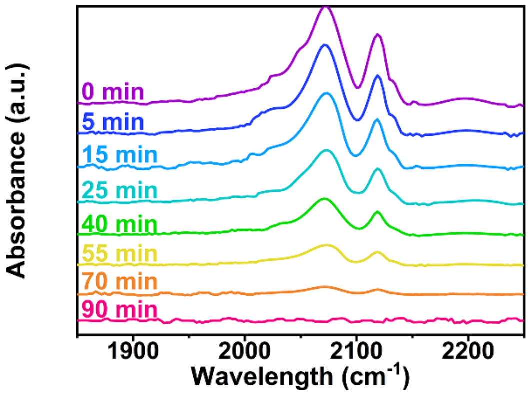

Supplementary Figure 34. DRIFTS spectra of CO desorption on ZnPor-Cu_2DAC under N_2 purging at ambient temperature and pressure.

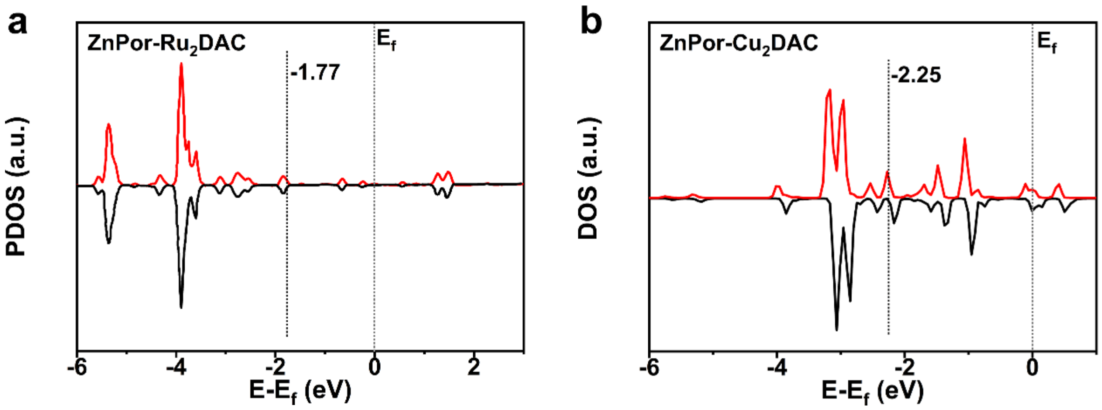

Supplementary Figure 35. Partial density of states with the band center energy marked for (a) Ru 4*d* of ZnPor-Ru_2DAC and (b) Cu 3*d* of ZnPor-Cu_2DAC.

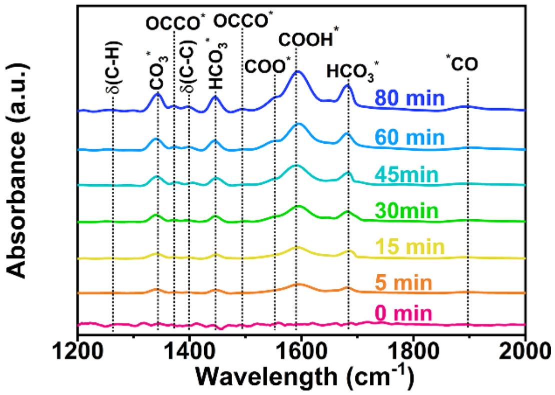

Supplementary Figure 36. DRIFTS spectra of CO_2RR on ZnPor-Ru_2DAC at different irradiation times.

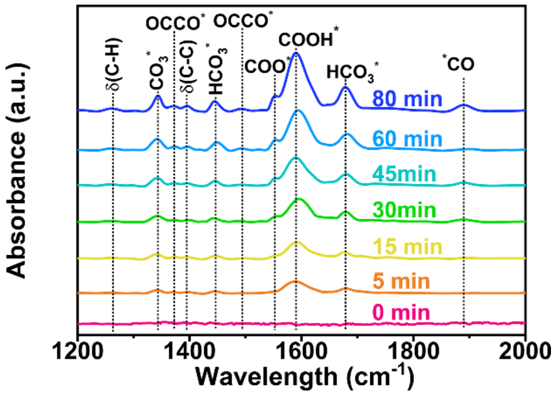

Supplementary Figure 37. DRIFTS spectra of CO_2RR on ZnPor-Cu_2DAC at different irradiation times.

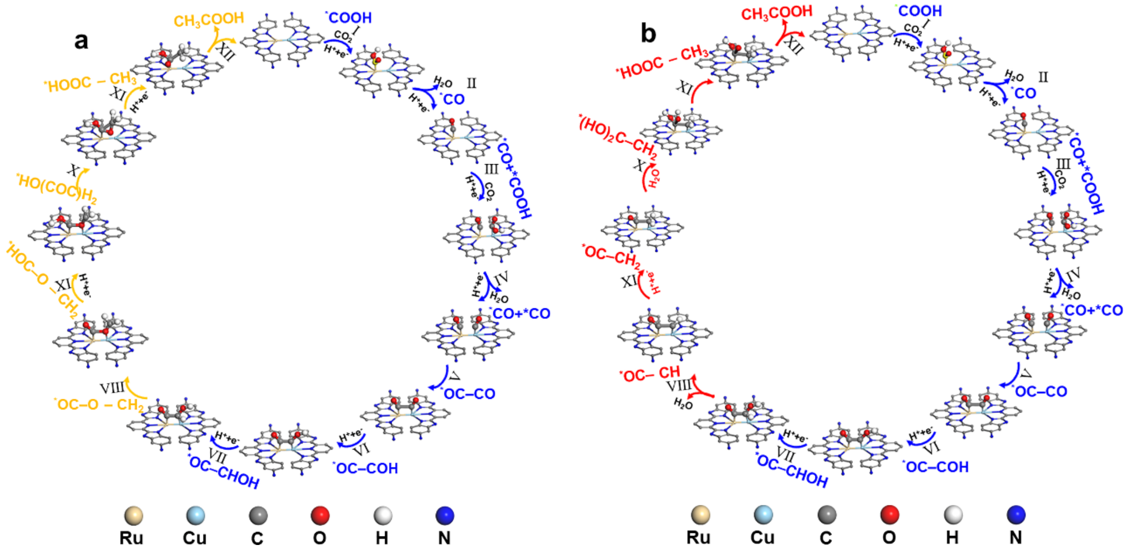

Supplementary Figure 38. The possible two reaction pathways during photocatalytic CO_2 reduction to acetate on ZnPor-RuCuDAC. For the produced CH_3COOH, (a) One of the O atoms afford from H_2O, and (b) both two atoms are offered from CO_2. The blue arrows are the same steps, while the green and red arrows present the different steps. Here, only a portion of the dual-atom sites is drawn to exhibit the intermediate configuration, even though the whole COF was selected for the calculation. The detailed steps are listed in the computational details section in the supplementary information.

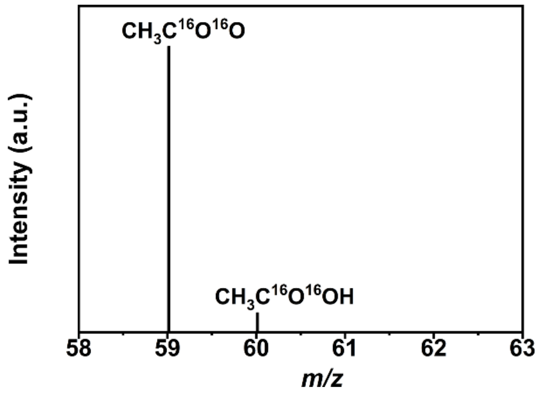

Supplementary Figure 39. Mass spectrum of acetate product with 10vol% $H_2{}^{18}O$ as proton source over the ZnPor-RuCuDAc CO_2RR.

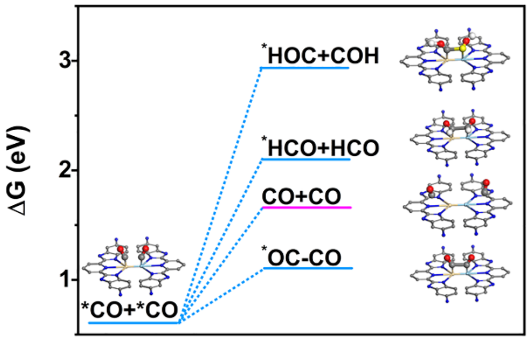

Supplementary Figure 40. The free energy changes for different reaction pathways of adsorbed *CO + *CO on the heteronuclear Ru–Cu site of ZnPor-RuCuDAC. The two *CO coupling energy barrier is 0.51 eV to form *OC–CO, the *CO desorption energy is 1.02 eV to form CO gas, and the Δ*G* of *CO protonation to (*HCO + *HCO) or (*HOC + *HOC) that can be protonated to CH_4, C_2H_6, etc is 1.56 eV or 2.28 eV, respectively. These Δ*G* values indicate that the *OC–CO intermediates, formed on heteronuclear Ru–Cu sites, are more thermodynamically favored than the *CO desorption and *CO hydrogenation to *CHO or *COH.

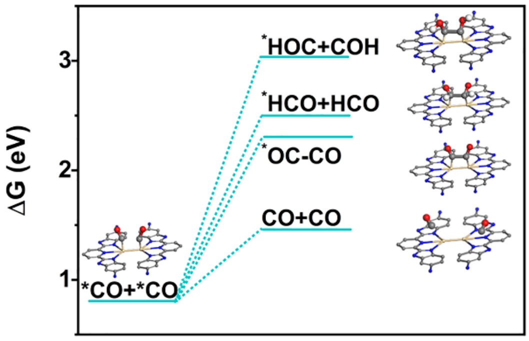

Supplementary Figure 41. The free energy changes for different reaction pathways of adsorbed *CO + *CO on the homonuclear Ru–Ru site of ZnPor-Ru_2DAC. The *CO desorption energy is 0.58 eV to form CO gas, the two *CO coupling energy barrier is 1.52 eV to form *OC–CO, and the Δ*G* of *CO protonation to (*HCO + *HCO) or (*HOC + *HOC) is 1.73 eV or 2.33 eV, respectively. These Δ*G* values suggest that the *CO desorption on homonuclear Ru–Ru sites is more thermodynamically favored than the *CO coupling and *CO hydrogenation to *CHO or *COH.

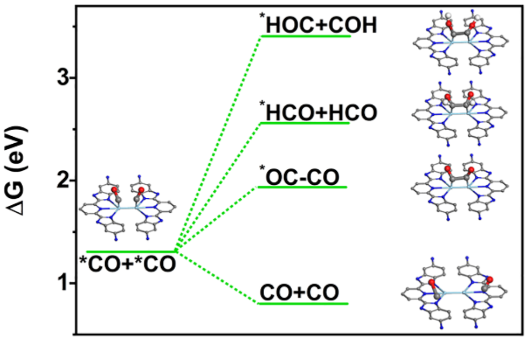

Supplementary Figure 42. The free energy changes for different reaction pathways of adsorbed *CO + *CO on the homonuclear Cu–Cu site of ZnPor-Cu_2DAC. The *CO desorption energy to CO gas is exothermic (–0.46 eV), the two *CO coupling energy barrier is 0.58 eV to form *OC–CO, and the Δ*G* of *CO protonation to (*HCO + *HCO) or (*HOC + *HOC) is 1.36 eV or 2.17 eV, respectively. These Δ*G* values reveal that the *CO desorption on homonuclear Cu–Cu sites is more thermodynamically favored than the *CO coupling and *CO hydrogenation.

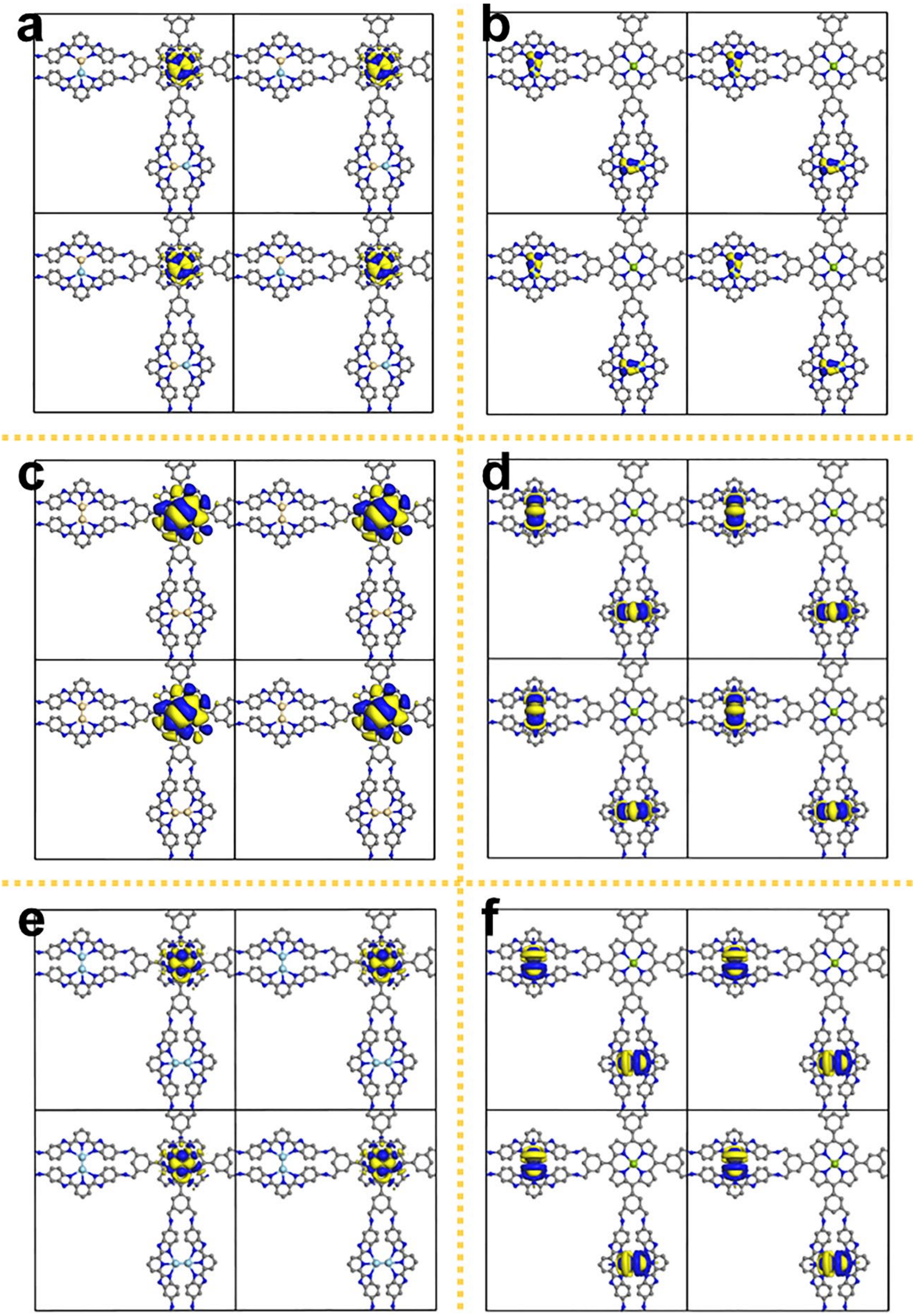

Supplementary Figure 43. Charge density distributions of ground-state (left) and excited-state (right) of (a,b) ZnPor-RuCuDAC, (c,d) ZnPor-Ru_2DAC, and (e,f) ZnPor-Cu_2DAC, respectively.

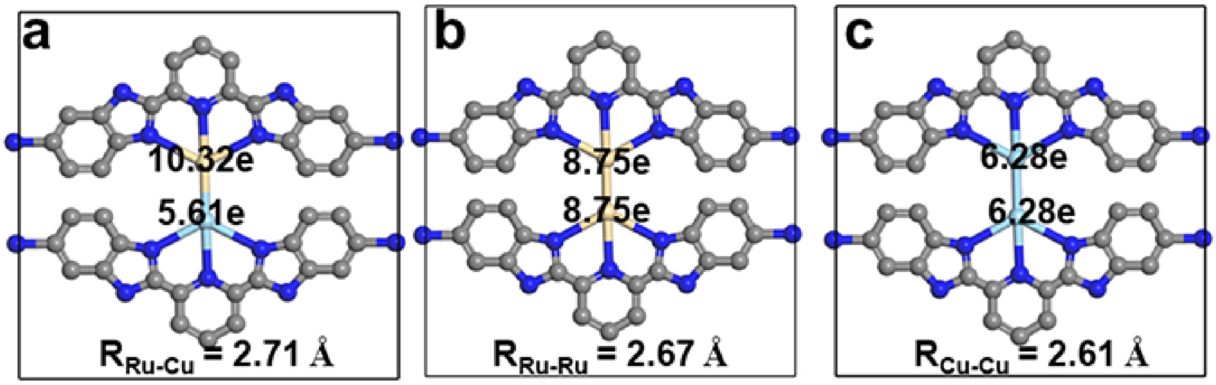

Supplementary Figure 44. The Bader charge values of different sites in the excited diatomic reactive sites of (a) ZnPor-RuCuDAC, (b) ZnPor-Ru_2DAC, and (c) ZnPor-Cu_2DAC, respectively.

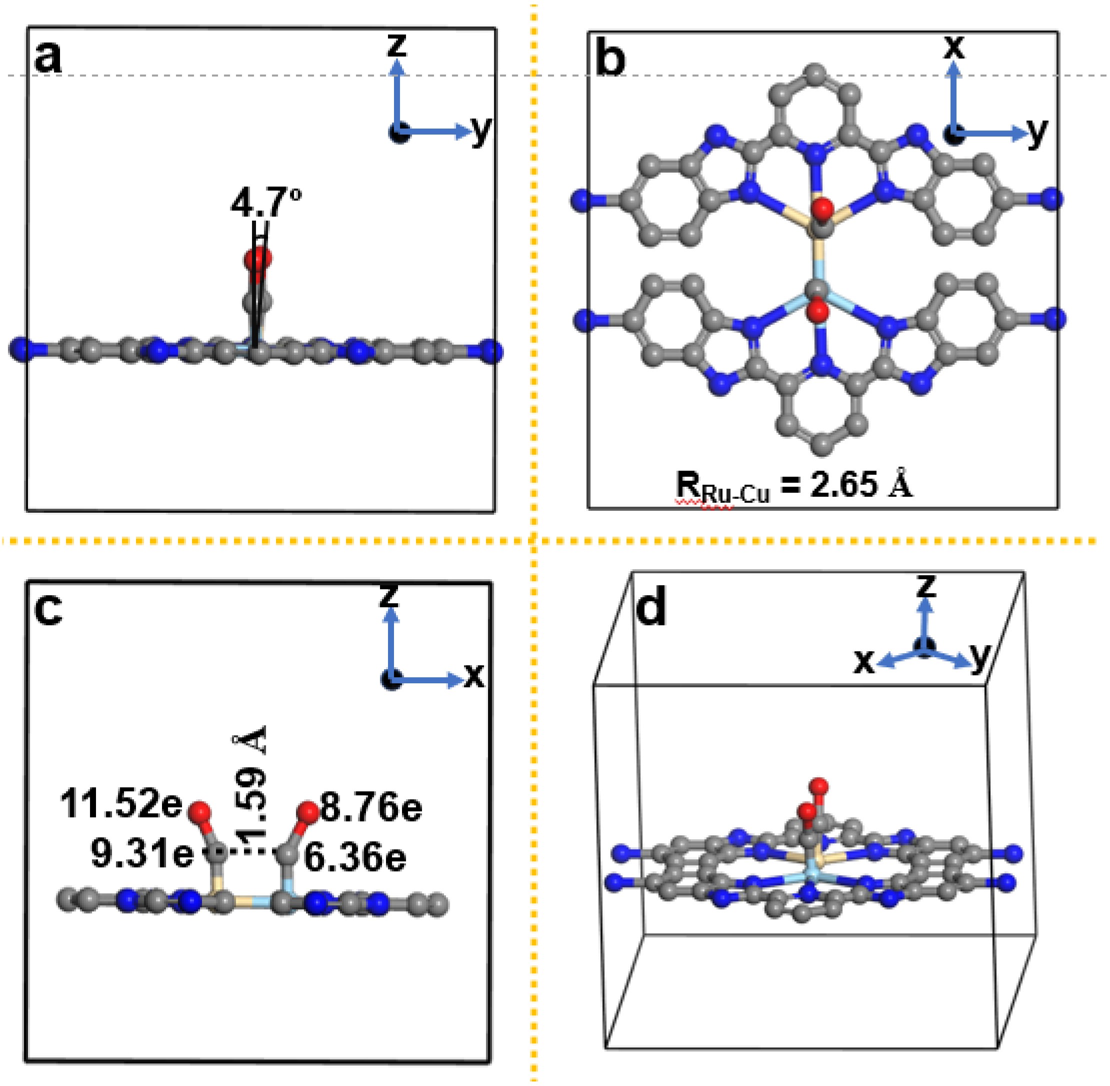

Supplementary Figure 45. Optimized structural parameters of two *CO on Ru–Cu site of ZnPor-RuCuDAC observed along the zy-axes (a), xy-axes (b), zx-axes (c), and 3D view (d), respectively.

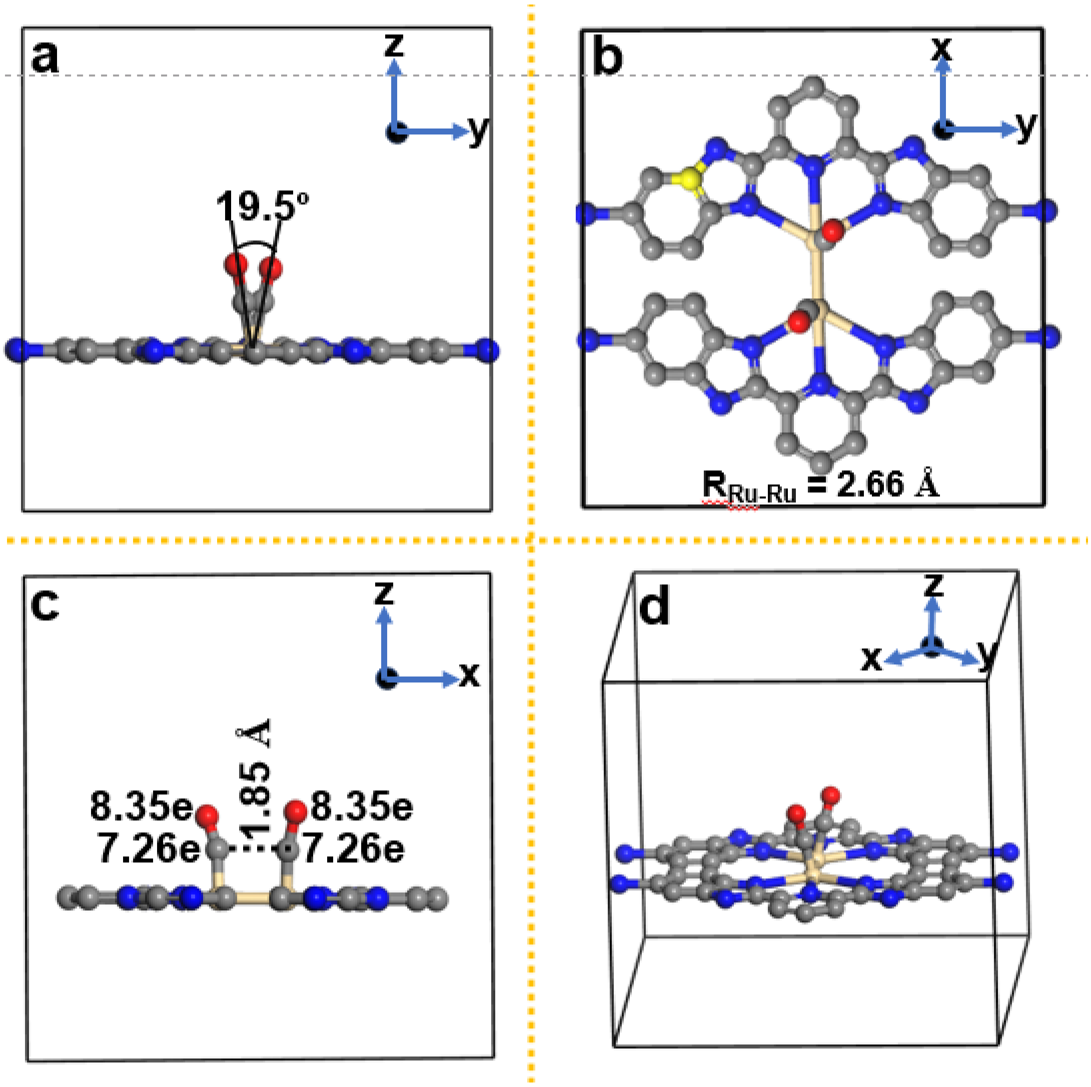

Supplementary Figure 46. Optimized structural parameters of two *CO on Ru–Ru site of ZnPor-Ru_2DAC observed along the zy-axes (a), xy-axes (b), zx-axes (c), and 3D view (d), respectively.

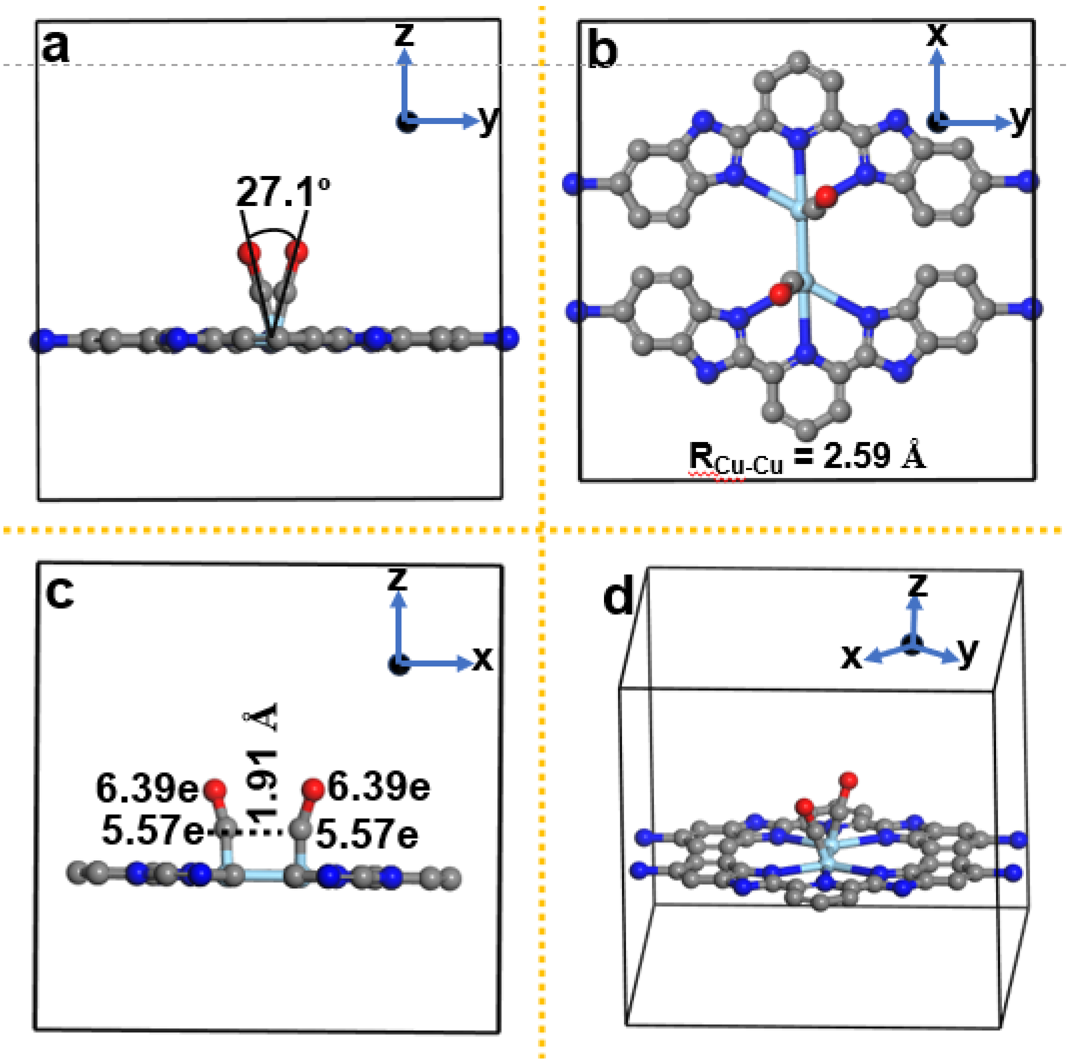

Supplementary Figure 47. Optimized structural parameters of two *CO on Cu–Cu site of ZnPor-Cu_2DAC observed along the zy-axes (a), xy-axes (b), zx-axes (c), and 3D view (d), respectively.

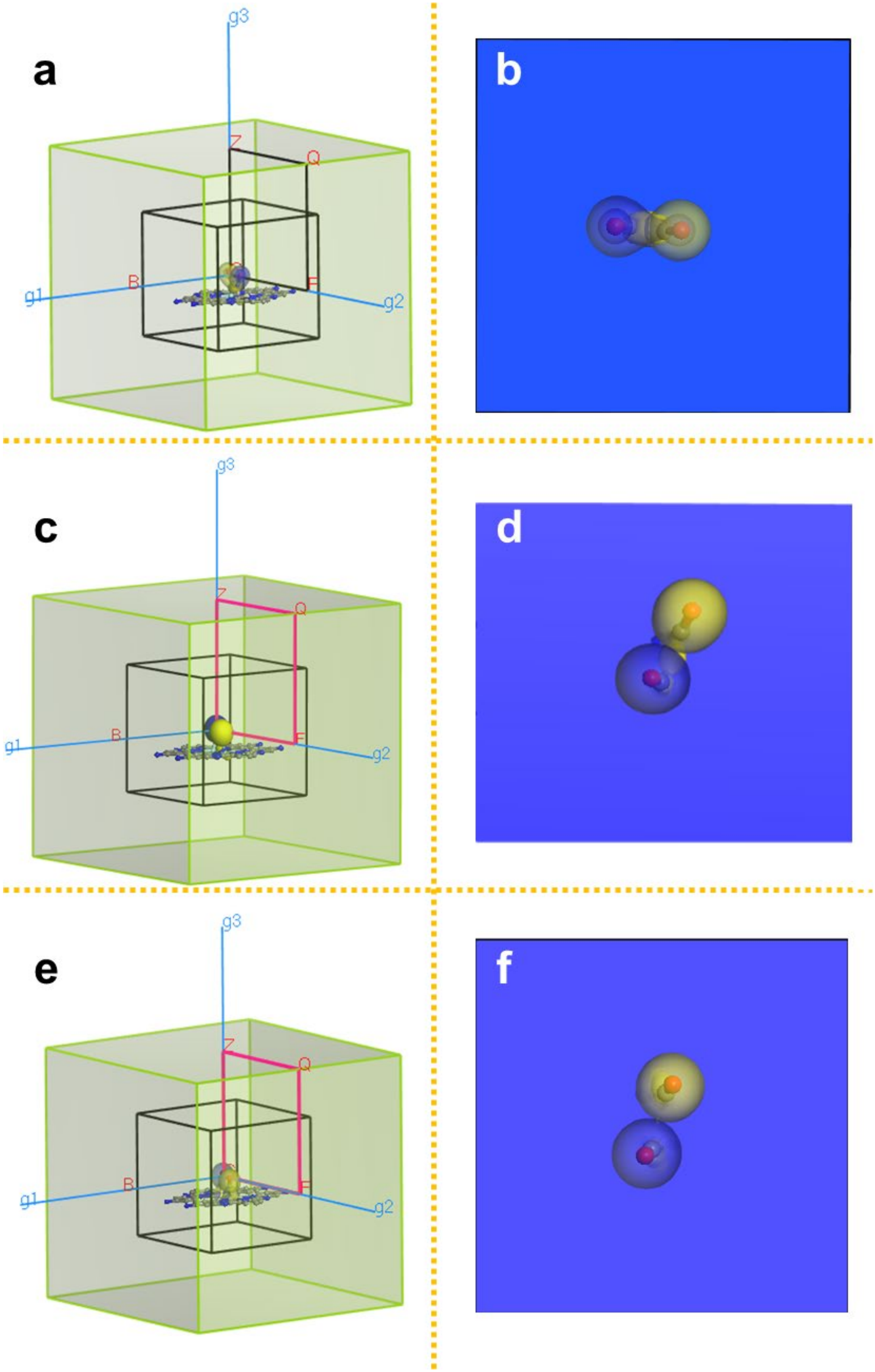

Supplementary Figure 48. Differential charge density maps of two CO* on the (a,b) ZnPor-RuCuDAC, (c,d) ZnPor-Ru_2DAC and (e,f) ZnPor-Cu_2DAC observed along 3D view (left) and y-axes (right), respectively.

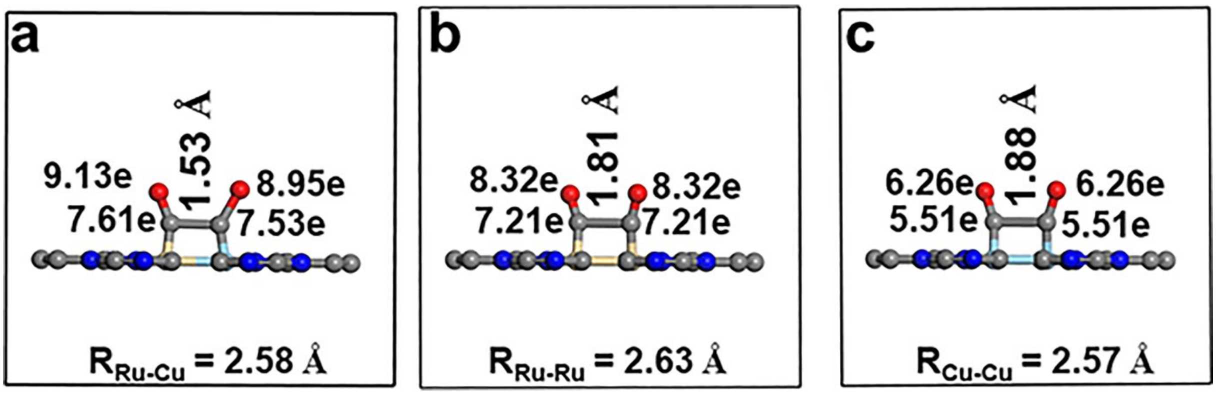

Supplementary Figure 49. Optimized structure of *OC−CO intermediate on diatomic site of (a) ZnPor-RuCuDAC, (b) ZnPor-Ru_2DAC, and (c) ZnPor-Cu_2DAC observed along y-axes, respectively.

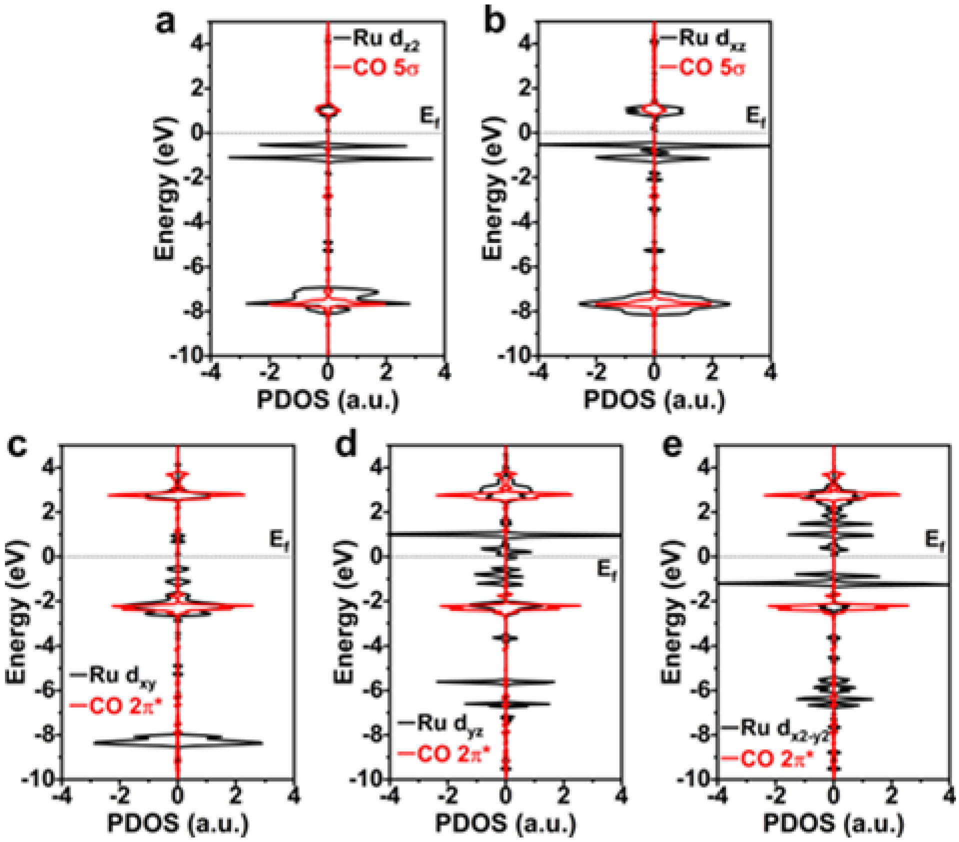

Supplementary Figure 50. Projected density of states of (a) (Ru d_{z^2}) – (CO 5σ), (b) (Ru d_{xz}) – (CO 5σ), (c) (Ru d_{xy}) – (CO 2π^*), (d) (Ru d_{yz}) – (CO 2π^*), and (e) (Ru $d_{x^2-y^2}$) – (CO 2π^*) on the ZnPor-RuCuDAC.

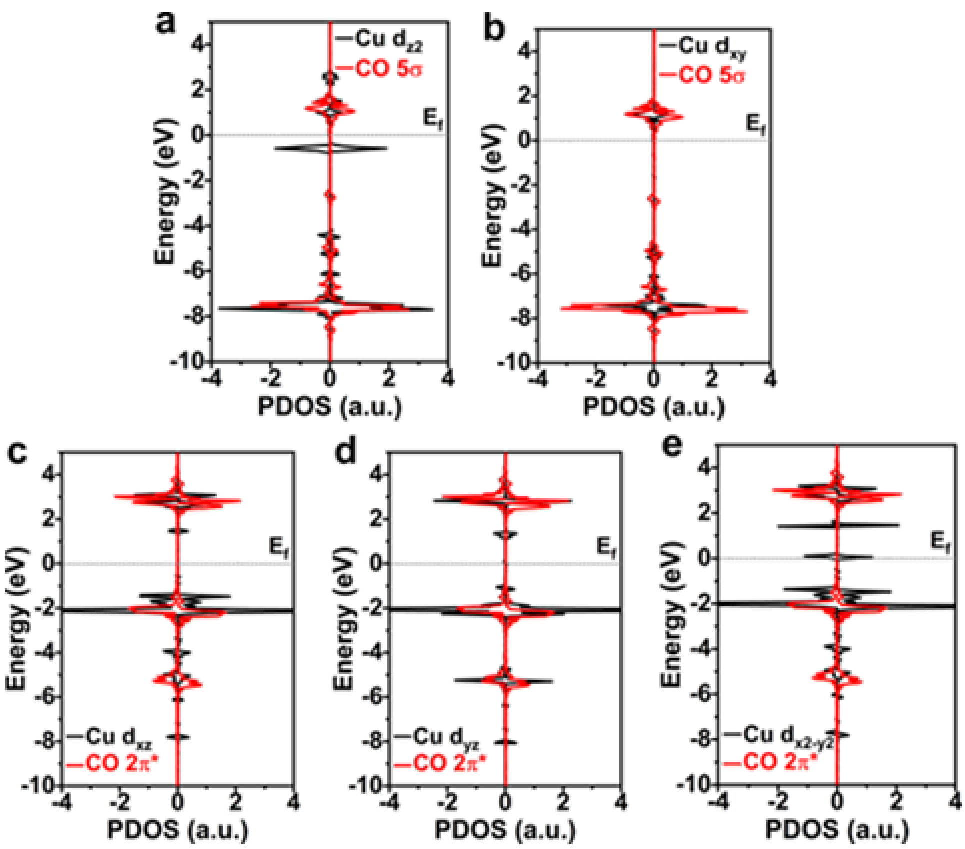

Supplementary Figure 51. Projected density of states of (a) (Cu d_{z^2}) – (CO 5σ), (b) (Cu d_{xz}) – (CO 5σ), (c) (Cu d_{xy}) – (CO 2π^*), (d) (Cu d_{yz}) – (CO 2π^*), and (e) (Cu $d_{x^2-y^2}$) – (CO 2π^*) on the ZnPor-RuCuDAC.

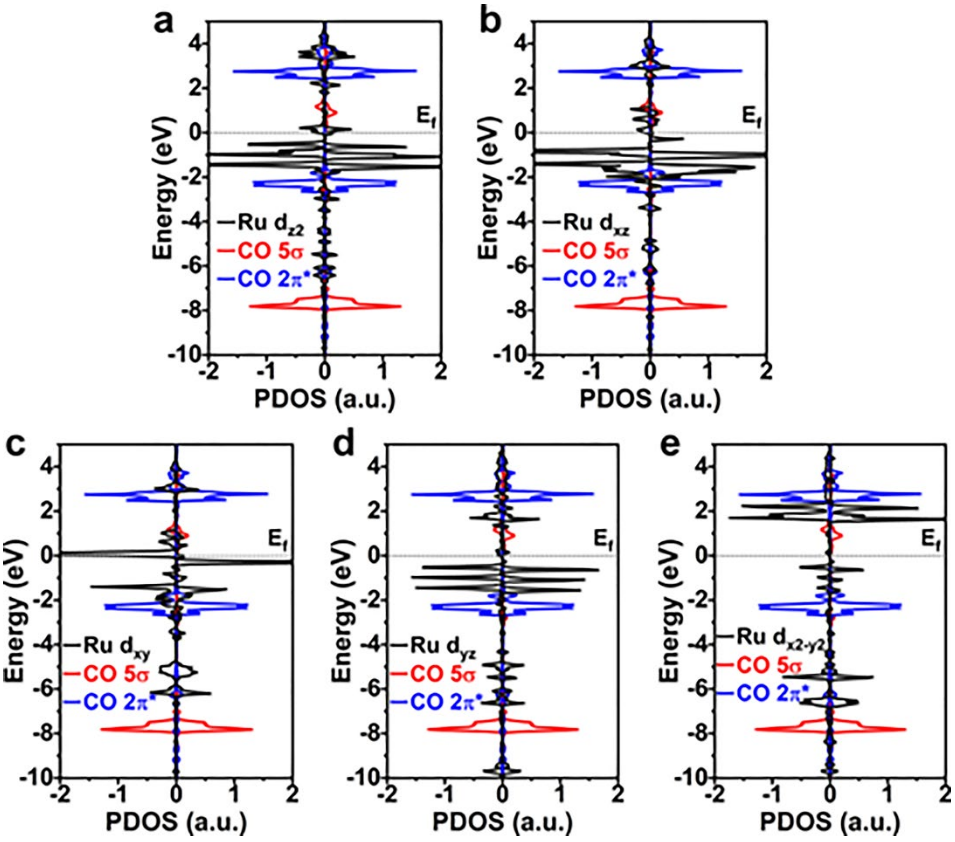

Supplementary Figure 52. Projected density of states of (a) (Ru d_{z^2}) – (CO 5σ), (b) (Ru d_{xz}) – (CO 5σ), (c) (Ru d_{xy}) – (CO 2π^*), (d) (Ru d_{yz}) – (CO 2π^*), and (e) (Ru $d_{x^2-y^2}$) – (CO 2π^*) on the ZnPor-Ru_2DAC.

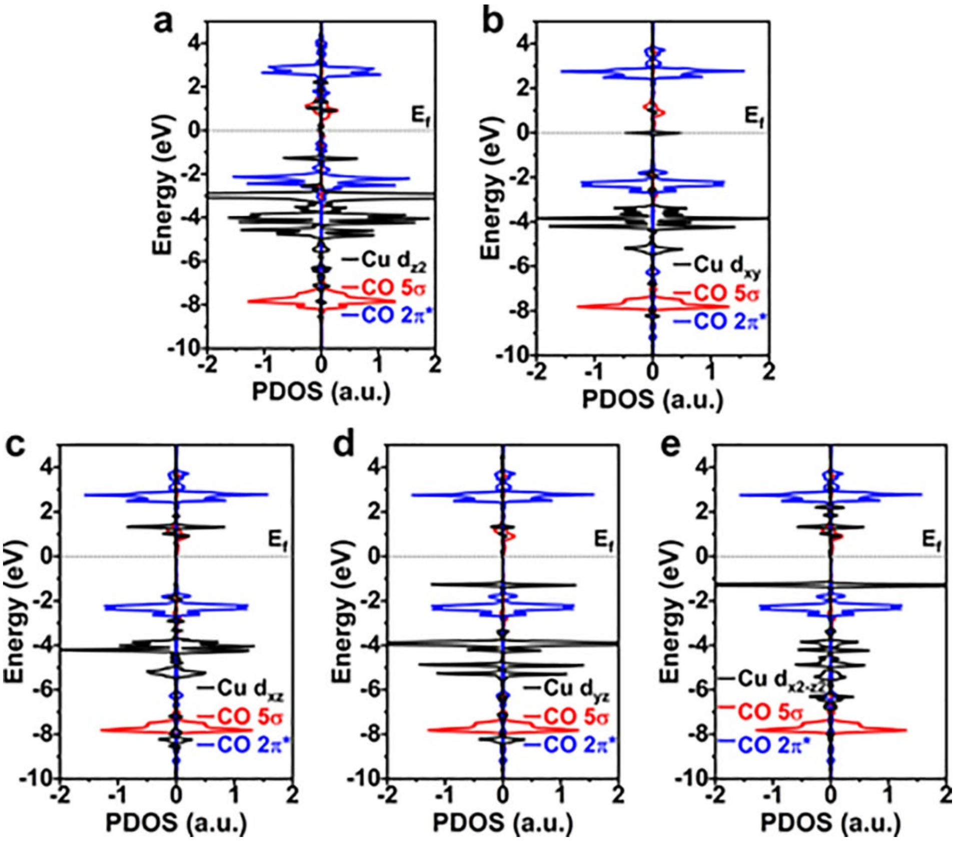

Supplementary Figure 53. Projected density of states of (a) (Cu d_{z^2}) – (CO 5σ), (b) (Cu d_{xz}) – (CO 5σ), (c) (Cu d_{xy}) – (CO 2π^*), (d) (Cu d_{yz}) – (CO 2π^*), and (e) (Cu $d_{x^2-y^2}$) – (CO 2π^*) on the ZnPor-Cu_2DAC.

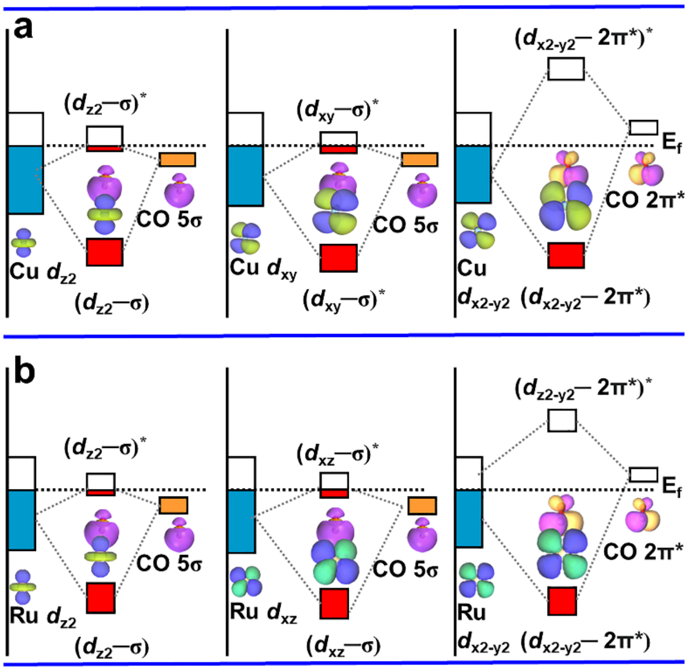

Supplementary Figure 54. Schematic illustration of adsorbed CO (5*σ*, 2*π**) orbital interactions with (a) Cu 4*d* orbital of ZnPor-Cu_2DAC and (b) Ru 3*d* orbital ZnPor-Ru_2DAC.

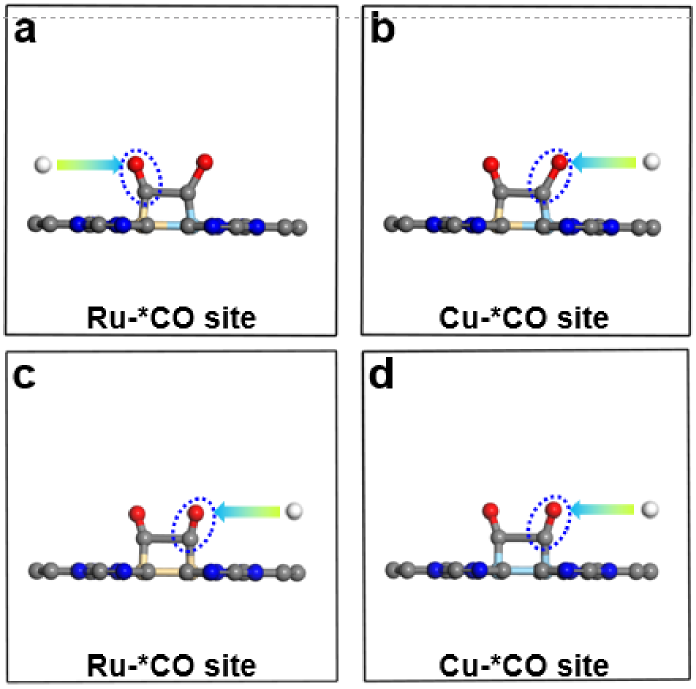

Supplementary Figure 55. Atomic configurations of *OC-CO intermediates adsorbed on the (a,b) ZnPor-RuCuDAC, (c) ZnPor-Ru_2DAC, and (d) ZnPor-Cu_2DAC, respectively. The blue circles presented a designated interaction for COOP calculations. The green arrows indicated the nucleophilic attack of proton species on OCCO* for the generation of OC-COH* intermediates.

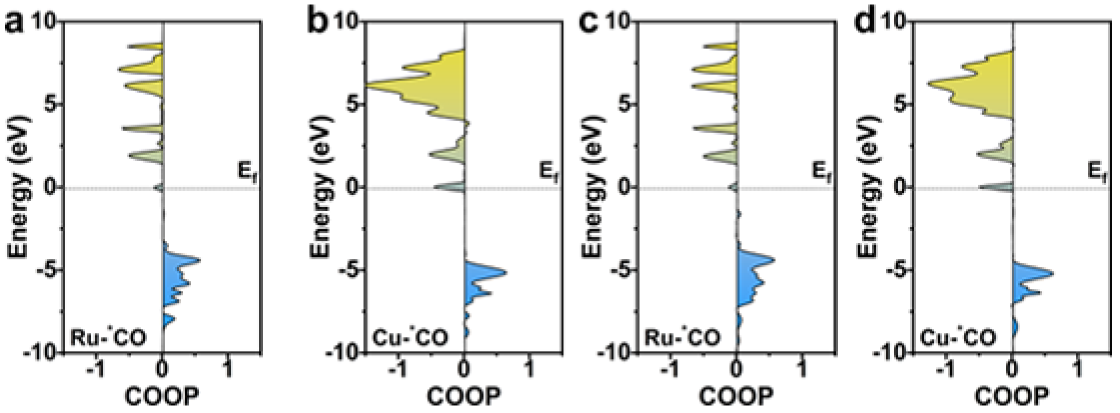

Supplementary Figure 56. Calculated crystal orbital overlap population between C and O atoms of *CO for *OC−CO adsorbed on (a) Ru and (b) Cu of ZnPor-RuCuDAC, (c) Ru of ZnPor-Ru_2DAC, and (d) Cu of ZnPor-Cu_2DAC, respectively.

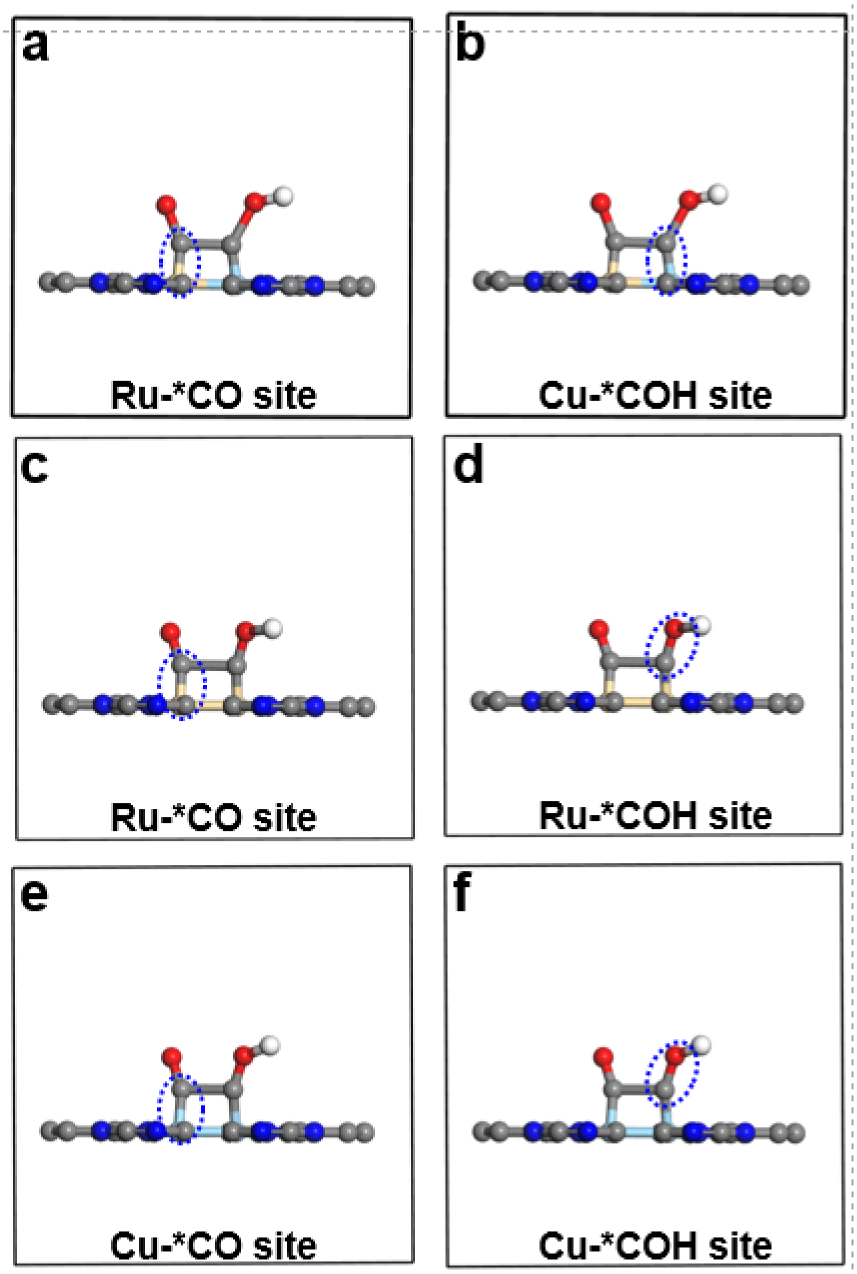

Supplementary Figure 57. Atomic configurations of *OC-COH intermediates adsorbed on the (a,b) ZnPor-RuCuDAC, (c,d) ZnPor-Ru_2DAC and (e,f) ZnPor-Cu_2DAC, respectively. The blue circles presented a designated interaction for COOP calculations.

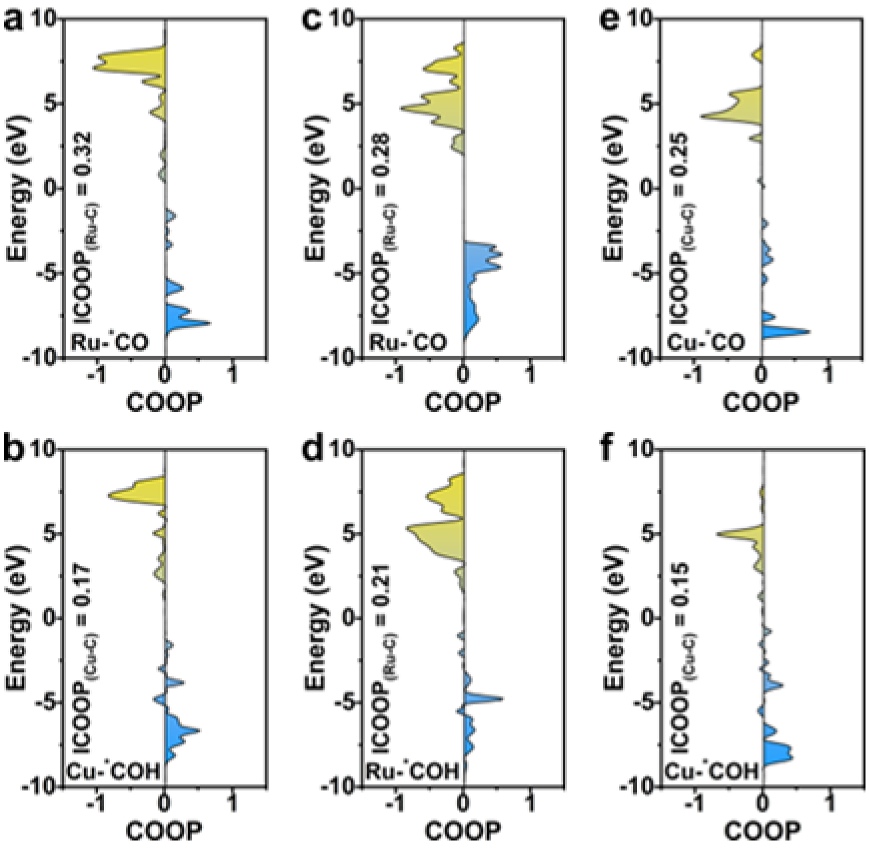

Supplementary Figure 58. Calculated crystal orbital overlap population between metal (Ru, Cu) and C atoms of *CO for *OC−COH adsorbed on the (a,b) ZnPor-RuCuDAC, (c,d) ZnPor-Ru_2DAC, and (e,f) ZnPor-Cu_2DAC, respectively.
